# Transcription factor clusters as information transfer agents

**Published:** 2024-03-05

**Authors:** Rahul Munshi, Jia Ling, Sergey Ryabichko, Eric Wieschaus, Thomas Gregor

**Affiliations:** 1 Joseph Henry Laboratories of Physics, Princeton University, Princeton, NJ 08544, USA; 2 Lewis-Sigler Institute for Integrative Genomics, Princeton University, Princeton, NJ 08544, USA; 3 Department of Molecular Biology, Princeton University, Princeton, NJ 08544, USA; 4 Department of Molecular Biology and Howard Hughes Medical Institute, Princeton University, Princeton, NJ 08544, USA; 5 Department of Stem Cell and Developmental Biology, CNRS UMR3738 Paris Cité, Institut Pasteur, 25 rue du Docteur Roux, 75015 Paris, France

## Abstract

Deciphering how genes interpret information from the concentration of transcription factors (TFs) within the cell nucleus remains a fundamental question in gene regulation. Recent advancements have unveiled the heterogeneous distribution of TF molecules in the nucleus, posing challenges to the precise decoding of concentration signals. To explore this phenomenon, we employ high-resolution single-cell imaging of a fluorescently tagged TF protein, Bicoid, in living fly embryos. We show that accumulation of Bicoid in submicron clusters preserves the spatial information of the maternal Bicoid gradient, and that cluster intensity, size, and frequency offer remarkably precise spatial cues. We further discover that various known gene targets of Bicoid activation colocalize with clusters and that for the target gene Hunchback, this colocalization is dependent on its enhancer binding affinity. Modeling information transfer through these clusters suggests that clustering offers a more rapid sensing mechanism for global nuclear concentrations than freely diffusing TF molecules detected by simple enhancers.

## INTRODUCTION

Transcription factors (TFs) play a pivotal role in regulating gene expression by interacting with DNA regulatory elements known as enhancers [1–3]. These enhancers often exhibit concentration-dependent behavior, activating or repressing gene expression only within specific TF concentration thresholds [4, 5]. The remarkable sensitivity of enhancers to subtle variations in the nuclear concentration of TF molecules implies that genes and enhancers carry out precise measurements of TF concentration [6, 7].

However, the challenge arises from the fact that in the nucleus, TF levels are often quite low and TF molecules are not uniformly distributed [8]. Instead, they assemble into dynamic transcriptional microenvironments referred to as transcriptional hubs [9–11]. These TF molecule accumulations are believed to form through transient clustering mechanisms [12–15] or through liquid-liquid phase separations (LLPS) , [16–18]. Separation of LLPS clusters reflects saturation kinetics, such that increasing concentration of the minor component results in increased size of droplets rather than an increase in the concentration within droplets [19, 20]. Whether droplet size provides a useful proxy for global nuclear concentration is unclear.

In this study, we aim to investigate whether these TF assemblies accurately reflect the nuclear concentration. We leverage the unique characteristics of the *Drosophila* TF Bicoid (Bcd), known for its varying concentration along the anterior-posterior (AP) axis of the early embryo [21]. Despite low nuclear concentrations, Bcd exhibits an extraordinarily reproducible profile, revealing precision in positional information comparable to the size of a single cell [22–24].

Various imaging approaches have unveiled that, similar to many other TFs, Bcd is not homogeneously distributed in the nucleus [25–27]. Instead, it forms numerous cluster-like droplets enriched with chromatin accessibility factors like Zelda [9] and actively transcribed canonical Bcd target genes, such as Hunchback, [28]. The higher concentrations within Bcd accumulations are believed to enhance transcription by increasing the local concentration near target enhancers [15, 29]. However, for these clusters to be functionally relevant to Bcd’s well-characterized role in patterning, some features of the observed clusters must convey positional information with a precision similar to the nuclear concentration profile.

To decipher which features of Bcd accumulations maintain information about concentration, we developed a rigorous quantitative imaging strategy. Contrary to simple LLPS models, we found that cluster size remains independent of concentration, while the cluster concentration varies linearly with nuclear Bcd concentration. These clusters localize at the locus of active target genes, conferring information about cellular position precisely. We use these data to explore quantitively the impact of clustering on information transfer and discuss the circum-stances where clustering might be a preferred mechanism as opposed to the gene interacting with the TF molecules freely diffusing in the nucleus.

## RESULTS

### Heterogeneity of nuclear TF distribution.

**FIG. 1.**
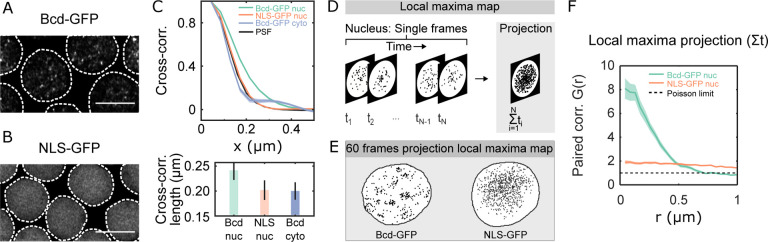
Quantitative characterization of nuclear Bcd heterogeneity. (A-B) Confocal (*Zeiss-Airyscan*) images of cross-sections of Bcd-GFP (A), and NLS-GFP (B) expressing blastoderm nuclei in living *Drosophila* embryos (NC14). Scale bars are 5μm. The broken lines represent a guide to the eye for the nuclear boundaries. (C) (TOP) Spatial cross-correlations computed on the nuclear pixels in 2D nuclear cross-section images expressing Bcd-GFP (green, 44 nuclei from 5 embryos) and NLS-GFP (orange, 27 nuclei from 3 embryos); and from pixels within the cytoplasm of Bcd-GFP expressing embryos (grey, 5 embryos). For comparison, the point-spread-function (PSF) of the objective is in black. Bottom panel shows mean and standard deviations of the computed correlation lengths: nucleoplasmic Bcd-GFP: 0.24±0.02μm; nucleoplasmic NLS-GFP: 0.20±0.02μm; and cytoplasmic Bcd-GFP: 0.20±0.02μm. (D) Schematic showing a map of local fluorescence intensity maxima inside a nucleus (left). The local maxima maps are extracted from individual frames of ~30 s long videos (60 frames) of nuclear cross sections (1μm thick). All maps from a given video are projected onto a single frame to form the local maxima map (right). See also METHODS and FIG. S3. (E) Representative local maxima maps for a Bcd-GFP nucleus (left) and an NLS-GFP nucleus (right). (F) Radial distribution function (or pair-correlation function, (r)) for the local maxima distribution expressed as a function of distance r from the center. G(r) was calculated on time-projected (60 frames each) local intensity maxima centroid maps, averaged over multiple nuclei (same nuclei and embryo counts as in C). A distinct peak in G(r) indicates temporally persistent confinement of the local maxima, as seen for Bcd-GFP expressing nuclei. For NLS-GFP, the gradual reduction in the radial function indicates a gradual decline in intensity near the nuclear edges without the existence of any sub-micron accumulations. (G(r)=1 corresponds to a perfectly uniform distribution).

We revisit the heterogeneous distribution of Bicoid (Bcd) within nuclei to establish quantitative insights. The distribution of Bcd within the nucleus comprises both freely diffusing molecules in intranuclear spaces and those engaging with chromatin [21, 30]. Cross-sectional images of Bcd-GFP nuclei (1μm thick z-section) revealed multiple focal accumulations per cross-section (FIG. 1A, FIG. S1A–D and FIG. S5). Conversely, embryos expressing an NLS-GFP fusion construct, where molecules diffuse freely without chromatin interaction, showed no such heterogeneity (FIG. 1B).

Quantitative analysis of the focal accumulations’ average sizes in these cross-sectional images, determined by pixel cross-correlation functions, revealed an average correlation length of 241 ± 17 nm for nuclear Bcd-GFP. In contrast, NLS-GFP expressing nuclei exhibited a smaller correlation length of 204 ± 16 nm, comparable to cytoplasmic Bcd-GFP (200 ± 17 nm) (FIG. 1C). Both nuclear NLS-GFP and cytoplasmic Bcd-GFP molecules are freely diffusing, and hence their correlation functions coincide with the microscope objective’s point spread function (PSF, FIG. S1F). Nuclear Bcd-GFP however, forms focal accumulations larger than the diffraction limit.

To investigate if the Bcd focal accumulations are spatiotemporally persistent, we took short videos of nuclear cross-sections. Local GFP fluorescence intensity maxima were identified in each video frame, and all frames were combined to form projection maps (FIG. 1D). The projection maps revealed that the Bcd-GFP maxima tend to crowd inside confinement areas within the nucleus, contrasting with the dispersed maxima in NLS-GFP nuclei (FIG. 1E). Pair-correlation analysis [31] indicated an effective radius ($\xi_{\text{pair}}$) of the confinement area for Bcd-GFP nuclei as 369 ± 49 nm (FIG. 1F). No such correlation was detected in NLS-GFP nuclei. Within a confinement area, the density of local maxima is approximately eight times higher than outside (FIG. S4); 60% of frames recorded over a 30-second interval contain at least one maxima within the area. This implies that local maxima persist over the recording interval providing a minimal estimate of their lifetime and positional stability.

These results indicate that Bcd accumulations form persistent sub-micron clusters within the nucleus. To assess the potential of these clusters in transferring information to target genes, we proceed to characterize their biophysical properties.

### Bcd cluster properties

To characterize the biophysical properties of Bcd clusters in 3D, we took an approach different from the maxima detection approach used in the previous section. Since any cluster should be at least the size of the PSF along the x-y plane ( >4 pixels), an x-y cutoff of 3 pixels should eliminate any spurious spots. Also, from the persistence data (FIG. S4) and FIG. S1)), we argued that for an imaging frame time of ~500 ms, a cluster should span across at least two consecutive z frames, given the frame thickness is less than the z PSF (Methods). Using this approach, we identify between 40 and 70 analyzable clusters per nucleus.

**FIG. 2.**
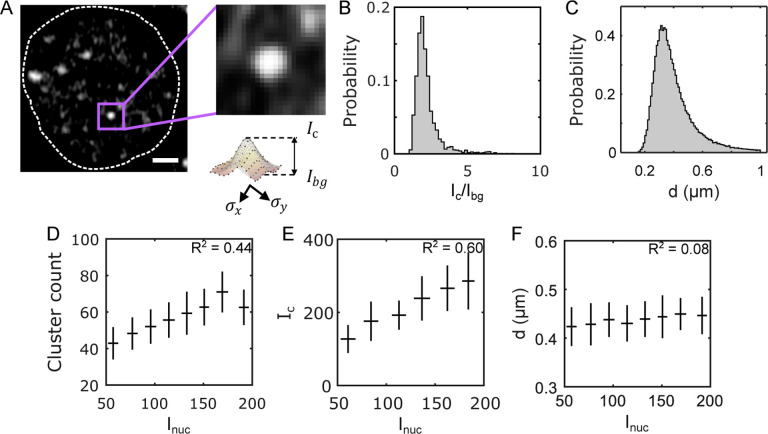
Biophysical properties of Bcd clusters (A) A single nucleus showing Bcd-GFP heterogeneities. Close-up image (right) shows a single Bcd-GFP cluster. Cluster intensity fit with a 2D Gaussian (see profile below). The cluster intensity $\left(I_{c}\right)$, the cluster background intensity $\left(I_{bg}\right)$, and the linear cluster size d are extracted from this fit (Methods). (B) A histogram of the signal-to-noise ratio ($I_{c}/I_{bg}$) and (C) a histogram of the linear size (d) for 99671 clusters from 2027 nuclei in 14 embryos expressing Bcd-GFP. (D) Number of clusters per nucleus as a function of average nuclear Bcd-GFP intensity (mean ± std). (E) Average cluster intensity per nucleus as a function of average nuclear Bcd-GFP intensity (mean ± std). (F) Effective cluster diameter (d) as a function of average nuclear intensity.

Individual cluster parameters are determined from 2D Gaussian fits of the GFP intensity profile on the z-plane of the cluster centroid (see Methods and Supplement). These fits yield estimates for the effective linear size of the cluster (d) of the clusters (Methods). The average linear size per nucleus is d=400±140 nm for all clusters (FIG. 2C). Notably, the histogram of the cluster size distribution reveals a vanishing left tail around the PSF limit, despite choosing a considerably smaller size cutoff than that limit. This implies that the detectable clusters are not diffraction-limited under our imaging conditions. It could be that the sub-diffraction clusters have very low intensities that are not fit for detection or are highly transient, rendering them undetectable. Either way, all such potential clusters would not be included in this study.

To gauge the cluster concentration, we introduce the parameter $I_{c}$, representing the cluster peak intensity, and $I_{bg}$), denoting the concentration of Bcd molecules in the nuclear space surrounding the cluster (FIG. 2A and FIG. S6). The signal-to-background ratio $I_{c}/I_{bg}$ offers insights into local Bcd concentration amplification within a cluster, with an average value of 2.2 ± 0.8 for close to 10^5^ clusters (FIG. 2B).

Given the variation of the nuclear Bcd concentration along the AP axis of the embryo (given by the average nuclear Bcd-GFP intensity, $I_{nuc}$), we investigate how cluster properties correlate with $I_{\text{nuc}}$. Analysis reveals a strong dependence of the cluster count on nuclear concentration, exhibiting an almost two-fold drop between the anterior and posterior poles (FIG. 2D, also see FIG. S7). This could result from a drop in $I_{c}$ and/or a reduction in d with decreasing $I_{\text{nuc}}$. From FIG. 2E and F, it is evident that while $I_{c}$ shows a strong dependence on $I_{\text{nuc}}$, with an almost two-fold change between the poles, d varies only insignificantly. This is further elucidated by the fact that the distribution of d effectively remains the same at various ranges of $I_{\text{nuc}}$ (FIG. S8B).

Thus, one might speculate that droplet growth by coalescence at higher concentrations, a characteristic of LLPS condensates, might be absent in Bcd clusters [32]. This speculation is complemented by the observation that the dependence of the cluster concentration $\left(I_{c}\right)$ on the nuclear concentration $\left(I_{\text{nuc}}\right)$ is linear $\left(R^{2}=0.6\right)$ (FIG. 2E), which contrasts with the switch-like dependence observed in liquid-liquid phase-separated condensates [20]. Notably, we observe clustering even in nuclei with very low Bcd concentrations, indicating the absence of a discernible threshold concentration triggering cluster formation [33]. However, further investigation is warranted to ascertain whether the clusters analyzed represent a matured state where conventional LLPS rules no longer apply, or if detailed imaging, capturing cluster formation dynamics is needed to distinguish between these possibilities.

### Do clusters contain enough positional information?

Previously, we have established that the position of anterior nuclei in the early *Drosophila* embryo can be determined with a spatial precision of better than 1 % from nuclear Bcd concentration alone [34]. This precision stems from the collective contribution of all nuclear Bcd molecules reproducible to within 10 %. Given that clusters comprise only a small fraction of molecules within nuclei (FIG. S11B), we sought to investigate whether they could offer an accurate estimation of nuclear concentration and, consequently, the position of the nucleus along the anterior-posterior axis of the embryo.

To this end, we consider a representative molecular count of Bcd within a cluster $\left(I_{m}=2I_{c}\sigma_{x}\sigma_{y}\right)$, where $\sigma_{x}$ and $\sigma_{y}$ are characteristic cluster fit parameters (see FIG. 2A). From this quantity, an absolute count for the total Bcd molecules within an average cluster can be computed using previous estimates for absolute molecular count conversions [21] (FIG. S11C).

**FIG. 3.**
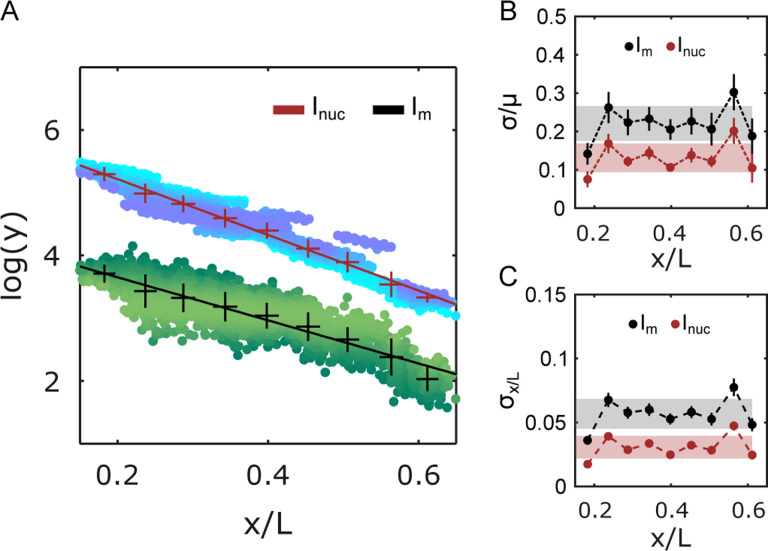
Precision of cluster positional information potential. (A) Natural logarithms of Bcd-GFP nuclear intensity ($I_{\text{nuc}}$, crimson), and total Bcd-GFP cluster intensity ($I_{m}$, black) are binned and plotted against binned nuclear position x/L. The mean and standard deviation shown in the error bars are obtained by bootstrapping (14 embryos, 2027 nuclei). Exponential decay constants extracted from linear fits (solid lines) were $\lambda_{I_{\text{nuc}}}=0.23\pm 0.01\;EL$, and $\lambda_{I_{m}}=0.21\pm 0.01\;EL$. Each point in the data cloud (shades of blue and green) represents a single nucleus. (B) Errors (σ) in $I_{\text{nuc}}$, and $I_{m}$, normalized to the respective means (μ) are plotted against nuclear positions. Gray and red shades indicate the overall error in position estimate across the entire axis. (C) The effective error in the estimation of the nuclear positions using $I_{\text{nuc}}$, and $I_{m}$ are plotted against nuclear position. Gray and red shades as in (B).

When computing the nuclear average from all $I_{m}$ in a given nucleus, this cluster-specific concentration decays exponentially with cell position as is typical for the Bcd gradient. The decay rate is comparable to the one of the overall nuclear Bcd concentration $I_{\text{nuc}}$, with a decay constant $\lambda_{I_{m}}=0.26\pm 0.02\;L$, which is statistically very similar to $\lambda_{I_{\text{nuc}}}=0.23\pm 0.03\;L$ (FIG. 3A). Thus, the molecular count of an average cluster mirrors the Bcd nuclear concentration gradient.

We calculated the corresponding variabilities (std/mean) across multiple nuclei located at identical spatial locations along the AP axis in multiple embryos for both $I_{\text{nuc}}$ and $I_{m}$ derived Bcd gradients (FIG. 3B). The overall variability in $I_{\text{nuc}}$ and $I_{m}$ were 14 ± 4 % and 22 ± 4 %, respectively. Despite the clusters representing only a small fraction of nuclear Bcd molecules (5–10 %, see FIG. S11), the $I_{m}$-derived Bcd gradient displayed remarkably low variability. This finding hints at the existence of tightly controlled mechanisms that regulate cluster formation.

As a morphogen, Bcd nuclear concentration imparts positional identity to a nucleus. To estimate the level of positional information contained in the cluster-derived Bcd gradient, we determine the positional precision σ(x) from the gradient’s concentration fluctuations δc(x) using error propagation [34]. Then the positional precision is given by $\sigma (x)=\delta c(x){\left|\frac{c(x)}{dx}\right|}^{-1}$, where c(x) is the Bcd concentration.

Using $I_{m}$ as the estimator for nuclear Bcd concentration, the positional error is c(x)=5.5±0.7%L (FIG. 3E), which corresponds to a positional precision of roughly three cell diameters. This is significantly worse than the previously shown single-cell precision obtained when using the full nuclear Bcd concentration $I_{\text{nuc}}$ as the estimator for the nuclear position. However, the estimation obtained from the cluster average $I_{m}$ reflects the property of an average cluster. Individual clusters might confer positional information with varying accuracy, with the highest potentially being equivalent to $I_{\text{nuc}}$. However, for genes to access this information, the clusters must be in physical proximity to specific gene loci.

### Cluster association with target genes.

**FIG. 4.**
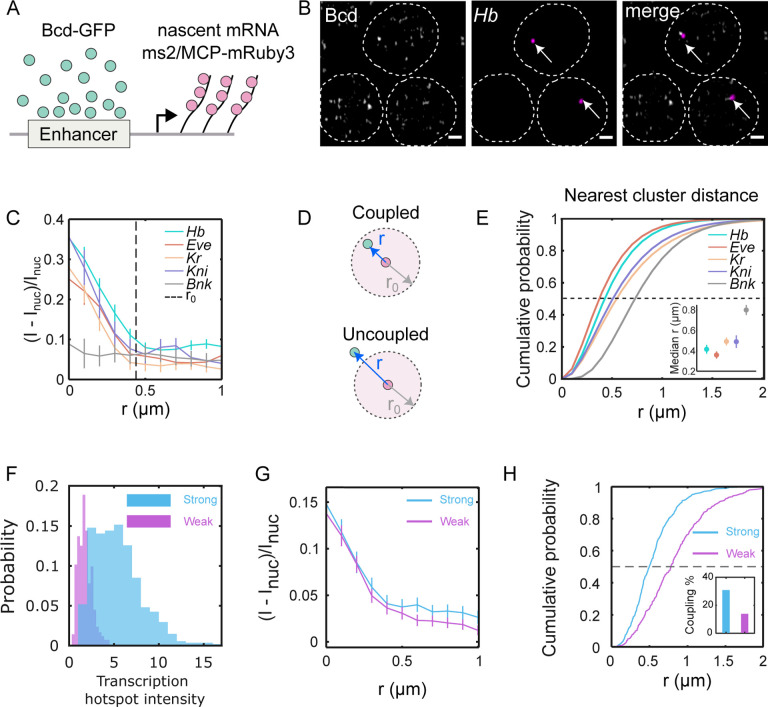
Bcd clusters co-localization with target genes is enhancer dependent. (A) Cartoon showing scheme for dual color imaging with Bcd-GFP (green) and nascent transcription site labeled via the MS2/MCP system (red). (B) Images from embryos in NC14 showing nuclei expressing Bcd-GFP and *hb*-MS2/MCP-mRuby on sites of active transcription (arrows); scale bar is 1μm. The dashed lines are guide to the eye for the nuclear boundaries. (C) Radial distribution of Bcd-GFP intensity with the centroid of the fluorescently labeled gene locus (i.e. hotspot) at the origin (see Methods). Data shown for canonical Bcd target genes, *hb, eve, kr, kni,* and the non-target gene *bnk*. Dashed line represents the average radius of accumulations from all genes combined $\left(r_{0}=0.44\pm 0.05\;\mu m\right)$. (D) Schematic showing the mRNA hotspot (red) and its nearest Bcd cluster (green). When the distance r between the nearest cluster and the hotspot is less than the Bcd accumulation radius $r_{0}$, the cluster is defined as being *coupled* to the gene; when it is greater than $r_{0}$ the cluster is assumed to be *uncoupled*. (E) Cumulative probability distributions of distances r for various genes; color code as in C. Dashed line determines median at EC50 (i.e. cumulative probability of 0.5). Inset: median distances for all genes. Errors are calculated from bootstrapping. (F) Histograms showing the transcription hotspot intensity from a strong and a weak enhancer construct driving an MS2-fusion reporter. The strong construct generates a 3.2-fold higher intensity, on average. (G) The radial distributions of relative Bcd-GFP intensity with the centroid of the transcription hotspot as the origin. The accumulation radii 0.36±0.05μm and 0.39±0.06μm for the strong and weak enhancer constructs respectively are statistically identical. (H) Cumulative probability distributions of distances r between the transcription hotspot and its nearest Bcd cluster. Black dashed line is at EC50. The median distances are 0.49±0.03μm and 0.78±0.05μm for the strong and weak constructs, respectively. Inset shows the fraction of coupled clusters for each construct (31 % and 13 %, respectively).

To elucidate the behavior of individual clusters around target gene transcription sites, we conducted three-dimensional imaging of labeled nascent mRNAs of putative target genes while imaging Bcd-GFP within the nuclei (FIG. 4A, B and FIG. S12). For each of the target genes (*hunchback, even-skipped, Krüppel, knirps*) [35, 36], Bcd accumulation was observed, with Bcd-GFP intensity peaks at the center of the nascent mRNA hotspot (FIG. 4C). The radii of Bcd-GFP accumulation around the four target genes were determined to be 485±39 nm, 546±23 nm, 394±44 nm, and 333±32 nm (FIG. S14A). These radii were comparable to the radius of the average enrichment area shown in FIG. 1F (see FIG. S13A for a simulation-based representation).

In contrast, Bcd accumulation was not detected around a non-target gene, *bottleneck* [37] (FIG. 4C). Nor does Bcd accumulate around the geometric nuclear centers, which are considered random sites that are unassociated with a particular gene locus (FIG. S13B). Confirmed specificity was attained by imaging NLS-GFP in place of Bcd-GFP, where no accumulation was observed around the *hunchback* locus (FIG. S13B).

The presence of Bcd accumulation near target gene loci indicates that Bcd clusters tend to have a high probability of colocalizing with the gene loci. However, a TF cluster may not be directly associated with the gene locus throughout the entire duration of active transcription of the gene locus. In such cases, the nearest TF cluster would be uncoupled from the gene (FIG. 4E), leading to a greater physical distance from the gene transcription site than a coupled cluster. The TF accumulation radius (FIG. S14A) gives a confinement radius within which a coupled cluster can be located. Utilizing this accumulation radius, a distance limit for cluster-gene coupling can be established, where any TF cluster located within that distance limit can be considered coupled to the respective gene.

The median 3D distances of the nearest Bcd clusters from the center of the genes (mRNA hotspots) were determined to be 418.6nm, 355.5nm, 500.5nm, and 485.8nm for *hunchback, even-skipped, Krüppel*, and *knirps*, respectively; and it was 804.3 nm for the non-target gene *bottleneck* (FIG. S14A). Applying the respective distance limits (FIG. 4D) to the cumulative probability plots of the nearest cluster distance distributions (FIG. 4E), we calculated the fraction of clusters coupled to the respective genes (an alternate technique yielding similar results is shown in FIG. S13C). The fractions of coupled clusters were 0.57, 0.73, 0.41, and 0.30, respectively, for *hunchback*, *even-skipped, Krüppel,* and *knirps* (FIG. S14B). Since an accumulation radius is not well-defined for *bottleneck*, no localization fraction could be determined for this gene.

These findings suggest that Bcd clusters tend to localize with target genes with a high probability. Additionally, we observed that localization is enhancer-dependent. When comparing a strong and a weak enhancer for the *hunchback* gene, the strong enhancer produces a much higher transcriptional output (FIG. 4F), even though the Bcd concentration at the site of transcription are similar for both (FIG. 4G). However, the Bcd clusters are closer to the transcription site (FIG. 4H) for the strong enhancer, which also has a higher fraction of clusters bound to the active target compared to the weak one (FIG. 4H, inset).

Given that Bcd clusters carry information about the nuclear position, genes can thus access this information by directly interacting with the clusters. However, genes can also interpret the same information by directly interacting with molecules diffusing in the nucleus. Thus, the question arises: why is clustering favored?

### Clusters are fast information sensors.

Nuclear Bcd concentration has to be interpreted by Bcd’s target gene loci to accurately extract positional information from the morphogen gradient and trigger a transcriptional response accordingly. The ability to sense diffusing TF molecules naturally depends on the effective size of the sensor. Whether the size is of the order of a binding site (3nm), an enhancer (50nm), or the entire locus is unclear. We know from previous estimations that if a binding site is a relevant size metric, then the measured readout precision needs to invoke spatial averaging across multiple independent sensors, i.e. neighboring nuclei [34].

**FIG. 5.**
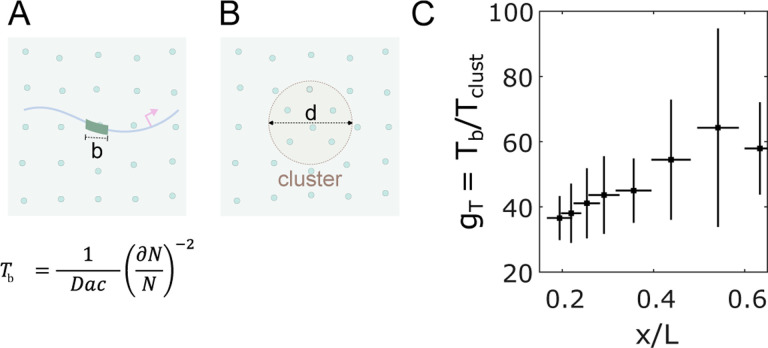
Clustering reduces time to precise concentration interpretation. (A) Two cartoons showing Bcd molecules in the nucleus (green circles) and an enhancer with a binding site of length b (left) or a cluster of diameter d (right) embedded in the nuclear environment. Below is the equation for the time taken by a sensor of size a for nuclear concentration c with an accuracy of $\left(\frac{dN}{N}\right)$, where N is the number of molecules counted. (C) Reduction of time $\left(g_{T}\right)$ to make an accurate (~ 10%) nuclear concentration estimation as a function of the nuclear position with the cluster as nuclear concentration sensor versus an enhancer binding site being the concentration sensor [34].

Here we consider the possibility that the TF cluster functions as a sensor and that the transcription output of a gene (its sensing of the gradient) is a reflection of the Bcd content of the cluster rather than interaction between single Bcd molecules at individual target gene enhancers. In previous analyses focused on enhancers, the time required for interpreting nuclear concentration was estimated using a molecular sensing argument that goes back to the work of Berg and Purcell [34, 38]. To apply this approach to Bcd’s heterogeneous distribution in nuclei, we treated a cluster as a sphere with an effective diameter d, the concentration of Bcd molecules inside the cluster as $c_{\text{clust }}$, and the diffusion constant of Bcd as D (FIG. 5B). The time $T_{\text{clust}}$ required for the cluster to precisely mirror the global nuclear concentration (the cluster sensing event that interprets nuclear concentration with an accuracy of $\left(\frac{\partial N}{N}\right)$) can be compared to the time $T_{b}$ required for a simple binding site in an enhancer of linear size b, to measure nuclear concentration (FIG. 5A).

The ratio $T_{b}/T_{\text{clust}}$ yields insight into the comparative sensing times. If clusters function as concentration sensors, the average cluster in an anterior nucleus in the embryo could sense nuclear concentration approximately 37.5±5.1 times faster than a single binding site (FIG. 5C), owing to the larger sensor size and ~ 2 fold concentration amplification within a cluster (FIG. 2B). Hence, an average cluster can interpret nuclear concentration in ~ 3 minutes, which is the timescale relevant to the activation of the target genes [34, 39]. In this scenario, no spatial averaging across neighboring nuclei or cells would be necessary. How Bcd content in the cluster interacts mechanistically with the target genes to govern transcription is unclear but points to an important area of future research.

## DISCUSSION

In this study, we employed quantitative imaging techniques in live embryos to elucidate the role of subnuclear compartmentalization, particularly clustering, in preserving the information carried by signaling molecules within the cell nucleus. Our findings highlight the potential significance of tight correlations between regulatory proteins and transcriptional outcomes, which may be mediated by transcription factor (TF) clustering within the transcriptional microenvironment. Notably, clustering facilitates the interpretation of concentration within transcriptionally relevant timescales, eliminating the need for invoking spatial averaging theories to explain the rapid regulation of transcriptional outputs [34, 40].

In such transcriptional microenvironments, various components such as mediators, chromatin-modifying agents, and PolII coexist and interact with each other. For instance, Bcd interacts with DNA via its DNA binding domain [41] and cooperatively with other proteins via its activation domain [42]. Our results suggest that the size of Bcd clusters is not solely dependent on the nuclear concentration of Bcd and that clusters can occur even at extremely low concentrations, contrary to classic liquid-liquid phase separation (LLPS) assemblies [19, 20, 27].

A more precise understanding of how Bcd clusters achieve nuclear concentration dependence should focus on the rate of approach of molecules at the cluster boundary, and how that rate is influenced by parameters relevant to diffusion [38]. Of these, parameters we assume that the diffusion constant and the surface area of clusters remain constant along the AP axis, leaving the nuclear concentration as the only variable. Once captured, the Bcd molecules reside within the cluster for a duration governed by their residence time before escaping [43, 44]. Cluster intensities will stabilize when capture rates are balanced by escape rates, thus allowing the final intensities of clusters to reflect overall nuclear concentration. Further theoretical exploration is warranted to elucidate the constraints on rate constants governing these processes.

Enhancers interact with TF molecules through binding events at DNA binding elements [1, 45]. Clustering amplifies local concentration, thereby increasing the probability of binding events at these sites and allowing enhancers to assay cluster concentration more rapidly. If Bcd’s diffusion within the cluster is similar to its general diffusion within the nucleus, the 2 fold increase in concentration in clusters will halve the measurement time at enhancers. To achieve the 37 fold increase calculated in FIG. 5, the cluster size must provide the important advantage and somehow the sensor function of the cluster must therefore operate at levels higher than simple enhancer binding. Enhancers dispersed along the genome communicate with gene promoters through DNA compaction mechanisms [46, 47], allowing them to share the same gene microenvironment as TF clusters [48]. This involvement of multiple enhancers in gene regulation adds complexity to the interpretation of nuclear TF concentration. Understanding how multiple binding sites within the same enhancer and the existence of multiple enhancers for the same target gene affect the measurement of nuclear TF concentration, and how those measurements are converted into transcriptional outputs, such as transcriptional bursts remains an open challenge.

Our analysis tests the feasibility of obtaining quantitative data on cluster properties from fluorescently labeled living embryos. Further studies should exploit this possibility to measure the lifetime of individual clusters and the dynamics of their association with target genes in both active and inactive states. Using systematically designed synthetic loci, one can also test the influence of locus complexity on concentration sensing, as well as cluster lifetimes. Understanding the information flow across length scales, from the comparatively larger scale of a cell nucleus to a gene locus from an information-theoretic perspective could yield valuable insights. In conclusion, While our work focuses on a particular TF in a model organism, the principles governing such clusters are likely to be applicable across diverse biological systems, including mammalian systems.

## MATERIALS AND METHODS

### Fly husbandry and genetics

*Drosophila* fly lines expressing bcd-eGFP from [49] were used as the starting point. In all such lines, the endogenous Bcd was replaced with a null phenotype $Bcd^{E1}$. Stable stocks expressing NLS-MCP-mRuby3 ; Bcd-eGFP-*bcd* ^E1^ were created. Virgins from these stocks were then crossed with males expressing reporter constructs with the gene regulatory regions, while the gene body was substituted with MS2 stem-loop cassettes and LacZ.

For the synthetic enhancers, the following scheme was used: A 472 base pair (bp) fragment spanning the modified *hb* proximal enhancer and the *hb* P2 basal promoter was synthesized by IDT and ligated into the piB-hbP2-P2P-MS2-P24x-lacZ-α Tub3’UTR construct [50] between the restriction sites HindIII and NcoI. In the resulting reporter construct the hb promoter drives the expression of 24 copies of the MS2 loops and is followed by the lacZ coding sequence. The number of MS2 loops in the reporter was verified by Sanger sequencing. In the strong enhancer reporter, 8 suboptimal Bcd binding sites were converted to the consensus sequence TAATCC, resulting in a total of 11 strong Bcd binding sites. In the weak enhancer reporter, all 3 consensus sequence TAATCC were converted to the suboptimal Bcd binding site TAAGCT, resulting in a total of 11 weak Bcd binding sites. Both constructs were integrated into the 38F1 landing site on chromosome II of the fly line FC31 (y+); 38F1 (w+) using FC31 integrase-mediated cassette exchange [51]. All fly lines from which males were crossed and their sources are tabulated in TABLE I.

### Sample preparation

Embryos were harvested on apple juice plates, using protocols mentioned earlier [49]. Staged two-hour-old embryos were dechorioned by hand by rolling them over a tape band (Scotch). Dechorioned embryos were placed on the lateral side on a mounting membrane lined with glue. The glue was prepared by submerging 10 cm of Scotch tape in 4 ml Heptane for 48 hours in a shaker at 37°C. A drop of glue was placed on the mounting membrane, gently smeared evenly, and was then allowed to air dry, before placing the dechorioned embryos. After the embryos were placed on the membrane, they were submerged in a mixture of halocarbon oil (60% Halocarbon27, 40% Halocarbon700, Sigma), and then covered with a 25 × 25 mm^2^ glass coverslip (Corning).

### Imaging

Three-dimensional stacks of fluorescence images were acquired using the fast airyscan mode of Zeiss LSM 880 microscope, run by Zen Black 2.3, SP1 software. A Plan-Apochromat 63x/1.4 oil immersion objective (Zeiss) was used for all measurements. For GFP excitation, 488 nm line of Argon laser was used (140μW), while mRuby3 excitation was done by a 561 nm diode pumped solid state laser (36μW). Laser power at the back aperture of the objective was measured with a Thorlabs power meter at the beginning of each measurement session. A MBS 488/561 beamsplitter was used to combine the beams. Two sets of emission filters were used: BP 420–480 / BP 495–550 was used for GFP emission, while BP 495–550 / LP 570 was used for mRuby3. The effective emission wavelengths were 515 nm for GFP and 578 nm for mRuby3. Detector gain of 740 was used for all imaging cases. The voxel size was fixed at 43 × 43 × 200 nm^3^ for all 3D measurements. For 2D single-plane videos, however, a z section of 1000 nm was used. The frame times were 497 ms for each frame for both color channels, with a pixel dwell time of 0.744μs. Each image frame was 1044 × 1044 pixels, or 45×45μm for the 3D acquisitions. No averaging was done. Imaging was done using the “Fast Airyscan” mode, and the final images were obtained after applying the “Airyscan Processing” within the Zen software.

Imaging was done on embryos in nuclear cycle #14, mostly from the 20th to the 35th minute after mitosis. The nuclei at the surface of the embryo facing the glass coverslip were imaged. A total z depth of ~14μm was imaged, split into 70 z-frames. This thickness was enough to scan entire nuclei, leaving some space at the top as well as the bottom. The z-stacks were imaged in four patches of 45×45μm for each embryo, at different positions along the AP axis. Each patch spanned across ~ 40 nuclei.

### Embryo fixation

The fixation procedure aims to retain the clusters while immobilizing them in space. Since we are dealing with near diffraction limit puncta with a low signal-to-noise ratio, it is best to avoid artifacts typically introduced by stained antibodies, which are usually used to visualize fixed samples. Hence, we aimed to use the fluorescence of the monomeric eGFP in Bcd-GFP expressing fly embryos to visualize clusters. This presents us with two challenges: 1) preserving the fluorescence of GFP after fixation, 2) preserving the clusters themselves.

We solved both issues using exclusively freshly dissolved methanol-free formaldehyde (to a final concentration of 4%) for embryo fixation and by minimal exposure to organic solvents like Heptane, Methanol, or Ethanol. With these modifications to a standard protocol [52], fixation and visualization of Bcd clusters in the embryos were achieved.

### Pixel cross-correlation

For this, the pixels along the x and y axes were separately autocorrelated with themselves. The crosscorr function of MATLAB was used for this purpose. This program uses the cross-correlation function, which is usually used to find the similarity between a time series and a lagged version of another. Here, we used either the same pixel row (x) or the same pixel column (y) in place of both the time series functions. Using pixel rows, (x), we get the correlation function (c) to be:

(1) $$c_{x,x}=\left\{\begin{array}{ll} \frac{1}{T}\sum_{t=1}^{T-k}\left(x_{n}-\overset{‾}{x}\right)\left(x_{n+k}-\overset{‾}{x}\right) & k=0,1,2,\ldots \\ \frac{1}{T}\sum_{t=1}^{T-k}\left(x_{n}-\overset{‾}{x}\right)\left(x_{n-k}-\overset{‾}{x}\right) & k=0,-1,-2,\ldots \end{array}\right.$$

This is repeated over all the rows and the average is then calculated. The columns are similarly treated and the average is calculated. The correlation lengths calculated along the rows or columns were the same for all images. The rows and the column data are then combined to obtain the overall image average. The average function was fitted with an exponential, y(x)=a+b⋅exp⁡(-c⋅x) and the “correlation length” was computed by $\lambda_{\text{corr}}=x_{0}+\frac{\text{log}(2)}{c}$. Subsequently, the error in the correlation length is given by, $\sigma_{\lambda }=\lambda \cdot \frac{\sigma_{c}}{c}$. This operation is selectively done for either the pixels exclusively within or outside the nuclear masks in the images.

### Local maxima detection

We locate local maxima in images of blastoderm nuclei indiscriminately, i.e. the resulting maxima may or may not represent a cluster. The process is broadly divided into two steps.

First, the nuclear pixels are selected and an Otsu thresholding is performed. Only the pixels above the threshold are retained and the rest are converted to “not a number”, (NaN). This removes the background pixels, leaving the candidates for maxima. A new set of μ and σ are calculated from the resulting non-NaN pixels. The image obtained is then rescaled to an interval [0, 1]. This process is repeated n times, iteratively to obtain a set of images $I^{i\in [1,n]}$.

In the second step of processing, local thresholding is done on the final image obtained from the previous step. For this, a filter window of 25 × 25 pixels is created. The image is then convolved with a normalized matrix, of the size corresponding to the filter window. This is done to apply a moving windowed mean $\left(\mu_{k}\right)$ filter on the image. Similarly, a moving standard deviation $\sigma_{k}$ filter is applied to the same image. From this, we can create a matrix of local thresholds of $\mu_{k}+\sigma_{k}$. Pixels under values determined by this local threshold matrix are set to zero. Thus a new image is obtained. The image is rescaled between [0, 1] and the process is repeated iteratively m times, so that we have a set of images $I^{\prime i\in [1,n],j\in [1,m]}$.

To determine the optimal m and n iterations, we calculate the structural similarity index (ssim) values, by comparing each binarized image $I_{bin}^{\prime i\in [1,n],j\in [1,m]}$ to the binarized image $I_{bin}^{\prime i\in [1,n],1}$. In-built MATLAB function ssim is used for this. The ssim value drops with each j∈[1,m], while the total “spots” detected does not significantly change. This is because each local thresholding operation retains a slightly smaller area around the local maxima, than the previous iteration. This also enables the splitting of two neighboring local maxima peaks after sufficient iterations. This is reflected in the positive change in the number of spots detected as the total spot area decreases. This iteration i,j sets the optimal thresholding criterion for spot detection.

Localization of the maxima is trivial from the final images and can be done with very high accuracy, by binarizing and using regionprops to find the maxima centroids.

### Pair auto-correlation

Consider randomly distributed points in space, described by Poisson process. In the same field, consider locations with significantly higher density of points which are also highly localized. This can be approximated by Gaussian point processes superimposed on a Poisson point process. To estimate the average density and effective size of these regions with Gaussian processes, we employ the pair correlation function [31].

The density function of the points expressed in polar coordinates is given by $\rho (\vec{r})$. We can calculate the pair correlation function for such a point distribution by [31]:

(2) $$g(\vec{r})=<\rho (\vec{R})\rho (\vec{R}-\vec{r}))>/\rho^{2}$$

Here, ρ is the average density. In practice, this correlation function can be calculated using Fast Fourier Transforms applied to an image I containing the point distribution.

(3) $$g(\vec{r})=\frac{1}{\rho^{2}}\cdot \frac{FFT^{-1}\left(|FFT(I){|}^{2}\right)}{FFT^{-1}\left(|FFT(W){|}^{2}\right)}$$

The I is a sparse matrix with 1 s at the locations of the maxima and 0 s elsewhere. The quantity, W is a window matrix adjusted to fit within the area of a nuclear cross-section taken as a convex hull.

The time projection of Bcd-GFP local intensity maxima has both randomly dispersed points as well as focal accumulations of points in space. The randomly distributed points can be considered to be representative of a Poisson process. The accumulations, however, can be considered Gaussian functions, convolved with hypothetical singularities. If we consider the density of the Poisson process to be 1, and the Gaussian process peak density to be $\rho^{\prime }$ above 1, we get the expression:

(4) $$g(r)=\rho^{\prime }\text{exp}\left(-r/\sigma^{2}\right)+1$$

Here σ denotes the size of the focal accumulation of the maxima, representing the Gaussian processes. This expression can then be used to fit the autocorrelation function to derive the effective width of the function as well as infer the increase in density within these Gaussian accumulations.

### Finding a nucleus’ position

To find the position of a nucleus in the embryo, a coordinate system relevant to the embryo needs to be defined first. First two images are acquired, one of the anterior and the other of the posterior ends of the embryo. The imaging is done on a single z-plane passing through the midsagittal plane of the embryo. The imaging conditions are kept the same as that of the nuclear images. This gives us the location of the anterior $\left(x_{0},y_{0}\right)$ and posterior $\left(x_{L},y_{L}\right)$ tips of the embryo (L signifying the length of the embryo) in the microscope stage coordinates. The line joining these two points represent the anterior-posterior (AP) axis of the embryo, and is the x-axis in the embryo co-ordinate system $\left(X^{\prime }\right)$. The perpendicular to this line, passing through $\left(x_{0},y_{0}\right)$ is the y-axis $\left(Y^{\prime }\right)$. Hence, the anterior end is (0, 0) and the posterior end is (L,0) in the embryo coordinates.

Now, if the centroid of a nucleus is $\left(x_{i},y_{i}\right)$ in the microscope stage coordinates, the distance of the nucleus from $\left(x_{0},y_{0}\right)$ is $r_{i}=\sqrt{{\left(x_{i}-x_{0}\right)}^{2}+{\left(y_{i}-y_{0}\right)}^{2}}$ and the angle made by $r_{i}$ with the AP axis is given by $\left.\theta_{i}=\text{arctan}\left(y_{i}-y_{0}\right)/\left(x_{i}-x_{0}\right)\right)-\text{arctan}\left(\left(y_{L}-y_{0}\right)/\left(x_{L}-x_{0}\right)\right)$. Hence, the location of the nucleus in the $X^{\prime }$ coordinates is given by $x_{i}^{\prime }=r_{i}\cdot \text{cos}\left(\theta_{i}\right)$, or:

(5) $$x_{i}^{\prime }=\sqrt{{\left(x_{i}-x_{0}\right)}^{2}+{\left(y_{i}-y_{0}\right)}^{2}}\cdot cos\left({tan}^{-1}\left(\frac{y_{i}-y_{0}}{x_{i}-x_{0}}\right)-{tan}^{-1}\left(\frac{y_{L}-y_{0}}{x_{L}-x_{0}}\right)\right)$$

This value can be computed for each nuclear controid. For pooling nucleus by position, nuclei with $x_{i}^{\prime }$ within the position bin edges are accumulated.

### Segmentatation of a “filled” nucleus.

Automated segmentation of Bcd-GFP expressing nuclei was done using the following scheme: First, the raw images were contrast adjusted using imadjustn, then filtered with a median filter, medfilt3, followed by a Gaussian filter, imgaussfilt3. A cuboidal structural element was then used for a series of morphological transformations to the resulting images. First, an erosion was applied (imerode), followed by a reconstruction, (imreconstruct), and dilation, (imdilate). The complement of the reconstruction transformation was then blurred with a Gaussian filter (imgaussfilt3). The resulting image was closed (imclose) and eroded. A binary mask for the nucleus pixels was subsequently obtained. Finally, watershed segmentation (watershed) was applied to separate any conjoined neighboring nuclei. Labels are then assigned to the nuclear masks.

### Locating clusters (in 3D)

A detection technique 2D local maxima localization was introduced earlier. This technique indiscriminately detects all local intensity peaks, which include noise spikes along with protein clusters. The difference between a “real” puncta and a “noise related local maximum” is that the spot size for a real puncta is at least as large as the point spread function of the microscope, while a noise related maximum is likely to be smaller. To capture this difference, we chose a pixel size which oversamples a diffraction limited spot. With an x-y size of 43 nm, a diffraction limited spot is spanned by 4–5 pixels in any direction. Hence, a cutoff limit of 3 pixels should effectively differentiate a real puncta from a noise related maximum. For z-slices, we chose a thickness of 200 nm. The PSF width along the z direction being larger than 500 nm, any maxima that is does not span atleast 2 z-slices is likely a noise related maxima. Hence we chose 2 pixels as the cutoff limit along the z axis.

At this point, one might point towards the fact that for live imaging of mobile structures (like subdiffusing clusters in our case), the likelihood of detecting a cluster in two consecutive frames is subject to the frame rate. To account for this, we refer to FIG. S4F, where we see that the detection probability of a cluster in frames imaged ~500 ms apart is > 70 %. Hence, for z-slices imaged ~500 ms apart with thickness less than the PSF width, we should be able to detect the cluster with a high degree of reliability.

To identify only relevant puncta-like entities in the nucleus, we employed the following technique on the raw images of Bcd-GFP nuclei. First, morphological top-hat filtering was applied using a “disk” as the structural element to the 3D raw images of the nuclei using imtophat. The transformed image thus obtained was used to detect local intensity maxima peaks. For this, the top 1 percentile pixels within a nucleus were selected from the transformed images. Joined neighboring spots were then separated by applying a watershed algorithm (watershed).

It must be noted that the “spots” thus detected are not the real spots, but rather peaks detected in the morphologically transformed image. However, it can be argued that the centroid of the local maxima peaks from the raw images are preserved through this transformation. Hence the detected “spots” can be used to locate the real cluster peaks. This information can be extracted by using the regionprops3 function. For this, the spot mask is obtained from the spot segmentation in the morphologically transformed image, and the mask is then applied to the raw image. Intensity weighted centroids in 3D of the voxels within each mask are then calculated using WeightedCentroid on the raw image. This gives the peak position of each cluster.

However, not all clusters thus detected are retained for further analysis. A size-based thresholding (as mentioned above) is then performed such that if the x-y cross section of a detected spot is less than 3 × 3 pixels wide and the z depth is not at least 2 pixels wide, the spot is discarded. That brings the threshold volume to 3 × 3 × 2 = 18 pixels. A corresponding effective spot diameter d can be calculated from the threshold volume, such that $d=(6/\pi \times \text{vol}{)}^{1/3}$. The threshold diameter turns out to be 3.25 pixels wide, which converted to absolute units gives, 138.2 nm, which is significantly less than the 3D PSF of the microscope. Thus, using this technique we identify the locations (only) of the “real” puncta in the Bcd-GFP nuclei.

### Fitting clusters

Cluster fitting is done on the raw image pixels to extract cluster relevant parameters like size and peak intensity. First, the plane corresponding to z coordinate of the cluster centroid is chosen and pixels within a square window with the cluster centroid as the center are extracted. The window length set at 2w+1 pixels, where w is ~12 pixels for a typical window size of $\sim 1\times 1\;\mu m^{2}$. A 2-dimensional Gaussian function is then used as the fitting function to fit the intensity profile within the window [53]. Fitting is done using lsqcurvefit, employing the levenberg-marquardt algorithm. The fitting equation is:

(6) $$f(x,y)=I_{p}exp\left(-\left(a{\left(x-x_{0}\right)}^{2}+\right.\right.\;\left.\left.2b\left(x-x_{0}\right)\left(y-y_{0}\right)+c{\left(y-y_{0}\right)}^{2}\right)\right)+I_{bg}$$

where,

(7) $$a=\frac{cos^{2}\theta }{2\sigma_{1}^{2}}+\frac{sin^{2}\theta }{2\sigma_{2}^{2}}\;b=\frac{sin2\theta }{4\sigma_{1}^{2}}+\frac{sin2\theta }{4\sigma_{2}^{2}}\;c=\frac{sin^{2}\theta }{2\sigma_{1}^{2}}+\frac{cos^{2}\theta }{2\sigma_{2}^{2}}$$

The pixel value at the center pixel of the window is used as the original guess for $I_{p}$, and a guess of $I_{bg}\approx I_{nuc}$ are used. The initial guesses for both $\sigma_{1}$ and $\sigma_{2}$ are w/2, while θ=0 is used for the rotational angle. The bounds for the σs are set to $w^{2}$ and the bound to $x_{0},y_{0}$ are set to -w:w. The bounds for θ is 0<θ<π/4. The cluster candidates whose fitting parameters do not satisfy the criteria are automatically discarded.

### Cluster parameters

We consider three cluster parameters here: cluster size, cluster peak intensity and cluster total intensity. An effective cluster size can be estimated from the σ obtained from the 2D Gaussian fit. While a cluster is a three-dimensional entity spanning over multiple imaging sections, we only do a two-dimensional fitting of the intensity profile of the plane passing through the intensity-weighted center along the z-axis. The imaging conditions are such that the resolution along z is significantly worse than the resolution along the x-y axes (worse by ~ 5 times). Therefore, any fitting along the z-axis will yield significantly higher error.

To calculate the effective size of clusters we consider the Gaussian spread along x and y directions $\left(\sigma_{x},\sigma_{y}\right)$. An expression for an effective radius ($r_{\text{eff}}$) can be obtained from these by:

(8) $$r_{eff}=\sqrt{\sigma_{1}^{2}+\sigma_{2}^{2}}$$

as the effective size of the three-dimensional cluster. In the above calculation, the Gaussian width is assumed to be a projection on a section of the obloid representing the real spot.

The peak intensity, $I_{c}$ is given by the peak intensity, $I_{p}$. The total intensity, or the area under the 2D Gaussian gives a measure for the total number of molecules in the clusters. The total intensity is given as:

(9) $$I_{m}=2\pi I_{c}\sigma_{1}\sigma_{2}$$

Hence, the expression for total intensity is independent of the expression for cluster size, even though it contains the expression for σs. Another thing to be noted is that $I_{c}$ and $I_{bg}$ are separately obtained from the fits, and hence the $I_{c}$ is automatically background corrected. Bounds of the fitting parameters are supplied and failure to return parameters within the bounds render the fits unusable, however, it is a rare occurence (< 2 %).

### Slope calculation

To calculate the properties of the average cluster in a nucleus, the nuclear average of the cluster properties are first calculated. The average data is pooled across all embryos and then plotted against the respective nuclear positions (x/L) or average nuclear concentration $\left(I_{nuc}\right)$. This data can be then divided into position or nuclear concentration bins. The concentration or the position bins were evaluated by fixing the total number of bins and accumulating data points within the bins by using histcounts. The mean and standard deviations of the nuclear average data per bin are found by bootstrapping. It needs to be emphasized that this does not reflect the standard deviation of the within a nucleus, but rather the standard deviation within a bin.

The average cluster properties like $I_{c},d$ and $I_{m}$ (or their respective natural logarithms) are plotted against the nuclear average concentration (or the nuclear positions). The data is then fitted with a linear regression model and the coefficient of determination, $R^{2}$, the slope of the linear fit as well as the error in slope are obtained from the fit parameters.

The cluster properties are assumed to linearly depend on the nuclear concentration, while the dependence with the nuclear position is assumed to exponential. Hence, the slope of the linear fit of the log of cluster properties plotted against the nuclear positions can be used to calculate the exponential decay constant: $\lambda =-(1/\text{slope})\pm \sigma_{\text{slope}}/\text{slope}{\;}^{2}.$

### Error in nuclear property estimation using cluster property

To calculate error in nuclear concentration estimation $\sigma_{c}$ using cluster properties like $I_{c},d$ and $I_{m}$, we use the slope obtained from the linear fits (s) and the error in the cluster property estimation in each concentration bin $\sigma_{i}$. We have $\sigma_{c}=\sigma_{i}/s$, while the error associated with each $\sigma_{c}$ is $\sigma_{\sigma_{c}}=\sigma_{c}\cdot \sqrt{{\left(\sigma_{\sigma_{i}}/\sigma_{i}\right)}^{2}+{\left(\sigma_{s}/s\right)}^{2}}$. Here, $\sigma_{\sigma_{i}}$ is the error associated with the cluster property determination and $\sigma_{s}$ is the error in slope. Similarly, the error in nuclear position estimation $\sigma_{p}$ using cluster properties can be computed by using the λ as follows: $\sigma_{p}=\lambda \cdot \sigma_{i}/i$, while the error in $\sigma_{p}$ is given by $\sigma_{\sigma_{p}}=\sigma_{p}\cdot \sqrt{{\left(\sigma_{\sigma_{i}}/\sigma_{i}\right)}^{2}+{\left(\sigma_{i}/i\right)}^{2}+{\left(\sigma_{\lambda }/\lambda \right)}^{2}}$. All these expressions are derived by using laws of error propagation.

### Empirical fitting function for cluster count

We can empirically arrive at a model to depict the concentration dependence of the number of clusters detected per nucleus. We base our model on the probability of a cluster being bound to the seeding site. This is given by $p_{on}=t_{on}/\left(t_{on}+t_{off}\right)$, where $t_{on}$ is the time for which the cluster is “bound”, and hence detectable, and $t_{off}$ is the time for which the cluster is “unbound”, and hence not detectable. To calculate these, we make three simplistic assumptions. 1. The total number of seeding sites in a nucleus is fixed, N. 2. The “bound” time, $t_{\text{on}}$ for a cluster i is a property of the seeding site only, n(i). 3. The “unbound” time, $t_{\text{off}}$ is a property of the diffusion parameters and is inversely proportional to the nuclear concentration, c, and hence to $I_{nuc}$. Therefore $\sum_{i=1}^{N}p_{on}(i,c)$ gives the total number of clusters detected per nucleus.

For the fitting function in FIG. S7E, N=80 has been used, and n(i) has been randomly generated.

### Segmentation of a “hollow” nucleus

Segmentation was also done to the MCP-mRuby3 expressing nuclei. Contrary to the Bcd-GFP expressing nuclei, the MCP-mRuby3 expressing nuclei are devoid of fluorophores and hence have a lower signal than the internuclear space. To segment such nuclei, the brightness of the images was first adjusted using imadjustn, and a three-dimensional median filter was applied. This was followed by a Gaussian filter and an extended maxima transformation was done (imextedndedmax). The resulting image was then binarized and the inverse of the resulting binary image was created. subsequently, a three-dimensional kernel was used to convolve the binary image. A threshold was then applied to the resulting image to which watershed segmentation was finally applied. the resulting image was opened with a cuboidal structural element, and any holes within the bright structures were filled to provide the final mask for the nuclei.

A “hollow” nucleus can be mapped to a “filled” nucleus if the corresponding centroids lie within a distance of less than half a nuclear length from each other. If the “hollow” and the “filled” masks cannot be mapped onto one another, the nucleus is discarded.

### Transcription hotspot detection

The transcription hotspots appear as distinctly bright spots in the nucleus against a significantly dark background. First, the nuclear bounds are found using the hollow nuclear segmentation scheme. To identify the transcription hotspots within the “hollow” nucleus, a Difference of Gaussian (DoG) algorithm was first applied to the raw images. The image obtained was then convolved with the raw image, and rescaled. A threshold based on the intensity of the nuclear pixels, $\left(I_{nuc}\right)$ was applied such that any pixels with an intensity $<\left(4\sigma_{I_{nuc}}+\mu_{I_{nuc}}\right)$ were discarded. Following this, the masks of potential transcription hotspots were obtained to which a size cutoff of 18 pixels was applied.

### Radial intensity profile and coupling fraction calculation

The intensity-weighted centroids of the transcription hotspots were obtained using regionprops after applying the transcription hotspot masks to the nuclear pixels in the MCP channel (mRuby3). The intensity of Bcd-GFP is calculated as a function of distance from the transcription hotspot centroid, $I_{r}$. For this, only the intensity along the x-y plane passing through the hotspot centroid is considered. $I_{r}$ is then obtained by averaging the values of pixels lying within the ring r and r+0.1μm. Data for each $I_{r}$ is pooled from multiple nuclei across many embryos to calculate the average $I_{r}$ profile.

The accumulation radius for a gene is calculated from its average $I_{r}$ profile. For this, the profile data is fitted with a double Gaussian function:

(10) $$f(x)=k_{0}e^{{\left(-\frac{\left(x-x_{0}\right)}{a_{0}}\right)}^{2}}+k_{1}e^{{\left(-\frac{\left(x-x_{1}\right)}{a_{1}}\right)}^{2}}$$

The accumulation radius $r_{0}$ is simply the FWHM width of the first of the two Gaussians and the error in radius is calculated from the fitting error. The distance of the intensity-weighted centroid of the closest Bcd-GFP cluster is measured from the centroid of the transcription hotspot. A histogram of all such distances is plotted and the cumulative probability function is calculated directly from the histogram or a spline fit to the histogram. The cumulative probability value at $x=r_{0}$ gives the coupling fraction for a gene.

Alternatively, the histogram of the nearest neighbor TF cluster distances from the mRNA hotspot can be fitted with double Gaussians. The first peak corresponds to the nearest neighbor cluster which is coupled to the gene, and the second, weaker peak is that of the cluster which is the nearest uncoupled cluster. The intersection of these two Gaussian fits marks the boundary $r_{0}^{\text{,}}$, such that only clusters inside this boundary are coupled.

The radius $r_{0}$ comes partially from broadening, as the chromatin is not stationary but rather subdiffusive in the nuclear space. This incorporates motion blurring of point sources during video capture. another source of broadening is the fact that the MS2 hotspot is not a point source itself. A hotspot consists of multiple MS2 stem loops, which are spread across the gene body and might span a few kilobases at any moment. Also, along with the nascent mRNA the stem loops project out of the gene body and have their degrees of freedom. Also, since transcription is a kinetic process, the stem-loops linearly traverse along the gene body with the speed of transcription (about 2kB/min). These factors combined are the biological source of broadening of the MS2 hotspot signal. Hence, the hotspot can be approximated as a point source convolved with a Gaussian that incorporates all forms of broadening.

### Estimation of molecules per cluster and the total cluster fraction

Estimation of the total Bcd molecules in the nucleus has been done in a previous study using Western blots [21]. However, the construct was such that the Bcd concentration was uniform throughout the embryo instead of decaying exponentially along the axis. However, since the total Bcd molecules in the case of the flat expressing line remain the same as the total expressing molecules in the wild-type gradient, we can arrive at a relation equating the two quantities as:

(11) $$N_{0_{\text{flat}}}\int_{0}^{L}dx=N_{0}\int_{0}^{L}e^{-x/\lambda }dx$$

Here, $N_{0_{\text{flat}}}$ is the average concentration of Bcd in a nucleus in the flat expression lines used in the study, while $N_{0}$ is the concentration at x=0 for the wild type line, expressing an exponential gradient with a length constant of λ.

Solving this, we get $N_{0}=N_{0_{\text{flat}}}L/\lambda$. Using $N_{0_{\text{flat}}}\sim 8000$, and λ~0.2, we get $N_{0}\equiv 40000$ molecules. Approximating an average nuclear diameter to 5μm, the average density of Bcd molecules in the nucleus turns out to be $600\;\text{molecules}/\mu m^{3}$. Considering that an average cluster is 2.2 times higher Bcd concentration than the nucleoplasm, the Bcd concentration inside a cluster at the anterior of the embryo turns out to be $1320\;\text{molecules}/\mu m^{3}$. The average diameter of a cluster is ~0.4μm, making the average volume, $0.03\;\mu m^{3}$. Knowing the molecular density within a cluster and the cluster volume, we can calculate the number of molecules per cluster as 37 in the anterior of the embryo. We show an estimate of the molecules per embryo along the embryo axis in FIG. S11C.

### Derivation of the concentration sensing limit

We consider the cluster to be a sphere with an effective diameter d, the concentration of Bcd molecules inside the cluster is $c_{\text{clust}}$, and the diffusion constant of Bcd is represented by D. The fractional error δN in counting N molecules by the cluster is given by

(12) $$\frac{\partial N}{N}={\left(\frac{6}{D\cdot d\cdot c_{\text{clust}}\cdot T_{\text{clust}}}\right)}^{1/2}$$

Rearranging EQ. 12 we get

(13) $$T_{\text{clust}}=\frac{6}{D\cdot d\cdot c_{\text{clust}}}{\left(\frac{\partial N}{N}\right)}^{-2}$$

The time, $T_{\text{clust}}$ in EQs. 12 and 13 represent the duration for which a cluster needs to “make the measurement” to interpret the nuclear concentration with an accuracy of $\left(\frac{\partial N}{N}\right)$.

The equivalent time a binding site of length a would take to measure the nuclear concentration is given by:

(14) $$T_{en}=\frac{1}{D\cdot a\cdot c_{nuc}}{\left(\frac{\partial N}{N}\right)}^{-2}$$

Here, $c_{nuc}$ is the nuclear concentration. Using EQs. 13 and 14 we obtain the ratio:

(15) $$\frac{T_{\text{en}\;}}{T_{\text{clust}\;}}=\frac{d}{6a}\left(\frac{c_{\text{clust}\;}}{c_{nuc}}\right)$$

The length of a typical binding site can be considered to be a=3.4nm [34]. However, $d,c_{nuc}$, and $c_{\text{clust}}$ are dependent on the nuclear position, making $T_{\text{en}}/T_{\text{clust}}$ a position dependent quantity.

### The point spread function

To compute the Point Spread Function of the microscope (PSF), 100 nm mounted fluorescent polystyrene beads (Thermo Fisher Catalog# T14792) were imaged with 1000 nm z-slice thickness with the Airyscan mode on Zeiss LSM 880 microscope. Pixel cross-correlation was done on the images. The correlation length gave the point spread function of the microscope.

## Supplementary Material

1
