# Supplementary material for: Transcription factor clusters as information transfer agents
Source: ArXiv. 2024 Aug 8:arXiv:2403.02943v2. Originally published 2024 Mar 5. Preprint. [Version 2] (PMC10942473)
Supplement: Supplementary file 1 [file NIHPP2403.02943V2-supplement-1.pdf]

I. SUPPLEMENTAL FIGURES

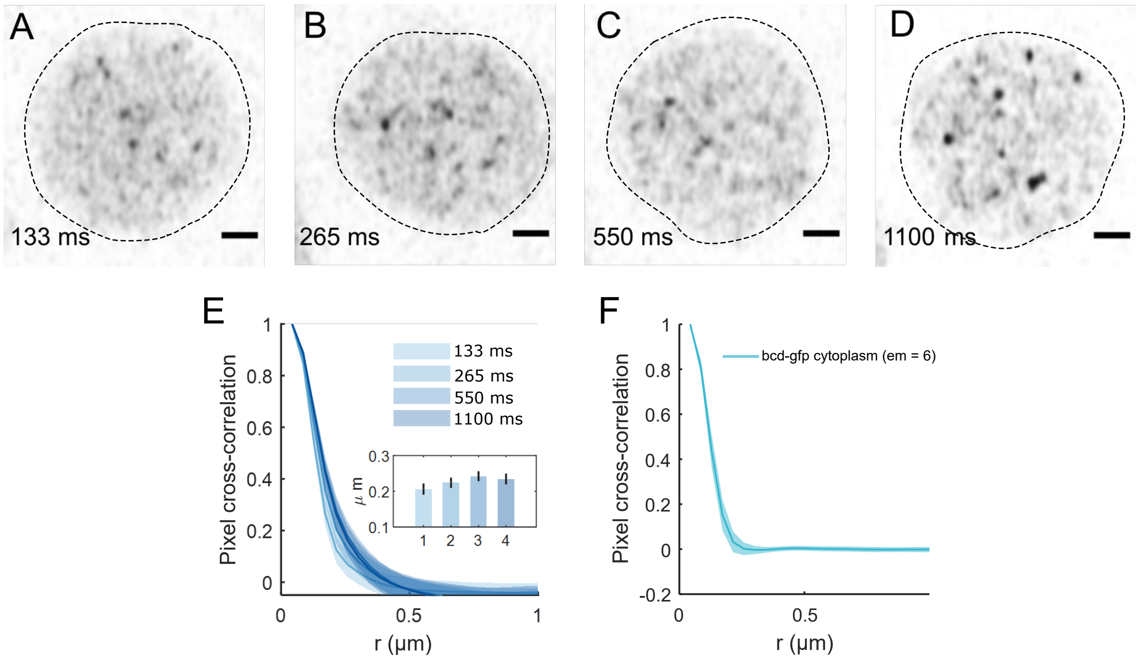

FIG. S1. **Imaging times and correlation lengths.** (A-D) Cross-sections passing through the centers of representative individual Bcd-GFP expressing nuclei, imaged at varying imaging times per frame (see annotation). Confocal *Airyscan* microscopy was used in the *fast* setting. Longer times per frame result in augmented mobile features blurring into the background, leaving only the brighter and more stable features visible (see increased contrast with 1100 ms setting). Dotted lines serve as guides to the eye for nuclear boundaries, and scale bars are 1 μm. (E) Pixel correlations are plotted for various times per frame, with the mean values and the standard errors shown as error bars in the inset. The correlation lengths are $0.21 \pm 0.02\ \mu m$, $0.22 \pm 0.02\ \mu m$, $0.24 \pm 0.02\ \mu m$ and $0.23 \pm 0.02\ \mu m$ for 133 ms, 265 ms, 550 ms and 1100 ms frame rates respectively. The correlation lengths are statistically indistinguishable, suggesting that motion-induced blurring does not significantly broaden resolvable structures, even at a time per frame of ~ 1 s. (F) Pixel correlation of cytoplasmic Bcd-GFP. We measure a correlation length of $0.19 \pm 0.02\ \mu$m, a value comparable to the width of the PSF, indicating that cytoplasmic Bcd mostly undergoes diffusive motion without forming clusters.

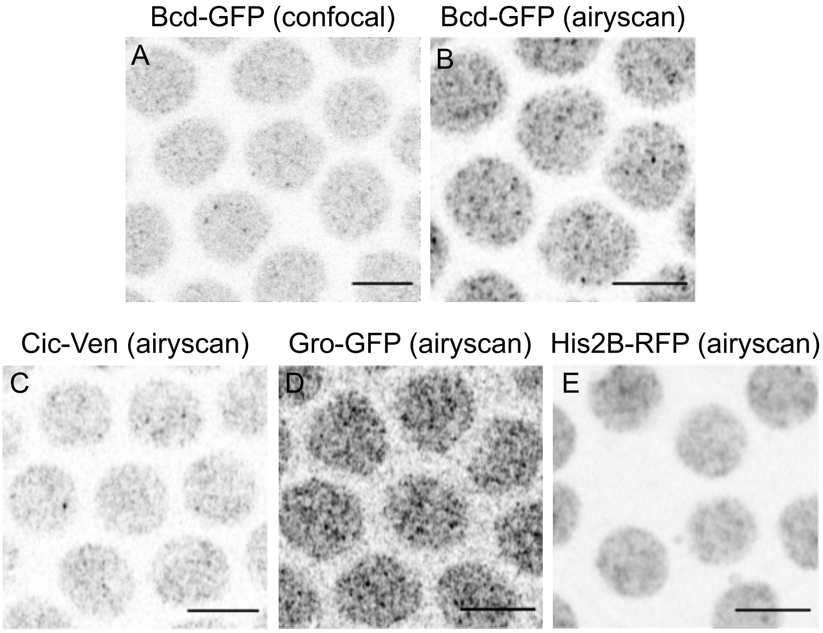

FIG. S2. **Various live protein labeling in *Drosophila* nuclei.** (A-B) Traditional confocal (A) as well as *Airyscan* confocal (B) images (Zeiss) showing cross sections of Bcd-GFP expressing nuclei in living *Drosophila* embryos during nuclear cycle 14. The pixel dwell times were the same for both images. Visual inspection identifies a higher signal-to-background ratio for cluster-like features in the *Airyscan* image than with regular confocal microscopy. This is an indication that the higher resolution of the *Airyscan* mode resolves smaller structures like the clusters better. (C-E) *Airyscan* confocal images showing nuclei expressing Capicua tagged with Venus, Groucho tagged with monomeric eGFP, and Histone2B tagged with RFP (otherwise imaging conditions as above). All scale bars are 5 μm.

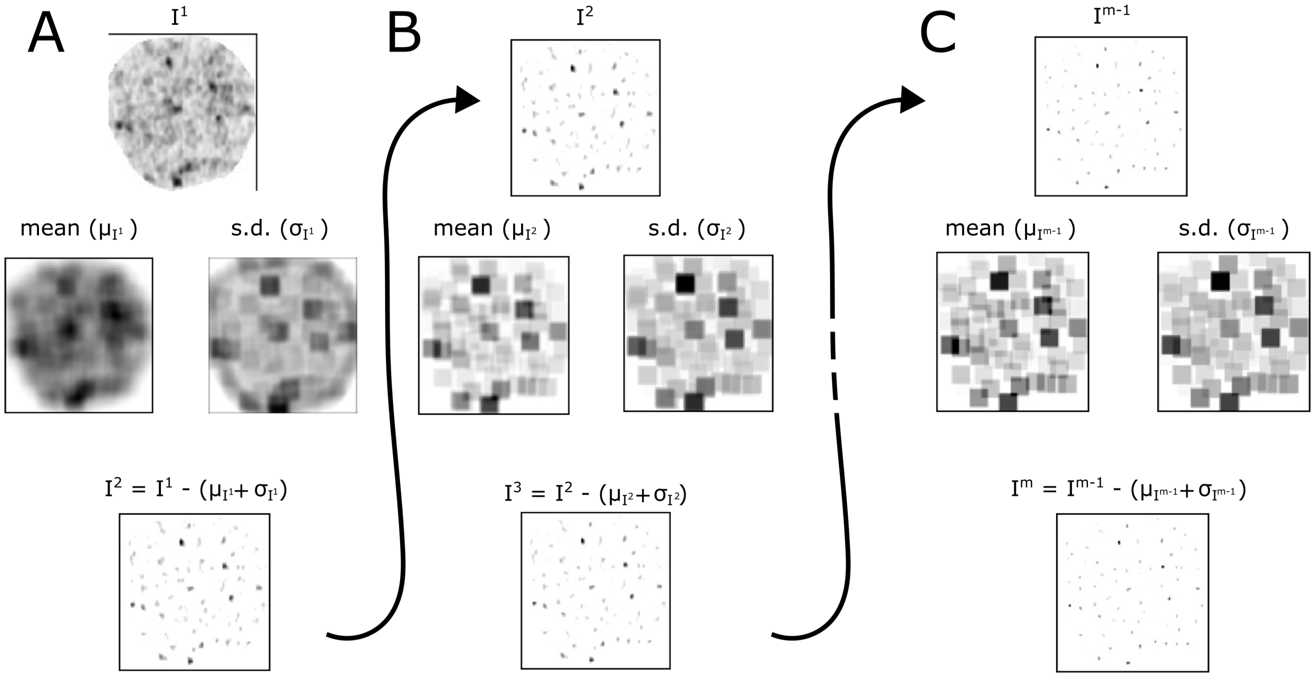

FIG. S3. **Local maxima detection algorithm.** (A) The top image displays the image of a nucleus expressing Bcd-GFP after Otsu thresholding. In the middle panel, the left image shows the moving means of the image at the top, computed over 25×25 pixel windows. The right image of the panel shows the moving standard deviation, over windows of 25×25 pixels computed on the image above. The bottom image is obtained by adding the middle row images and subtracting the resulting matrix from the top image. This resultant image is used for the subsequent operations in B. (B) The panel illustrates the recursive application of the process explained in A. This yields the image that acts as the input for the next layer of operations. (C) Finally, the image obtained after m successive application of local thresholding (Bottom) is utilized to locate the centroids of the local maxima.

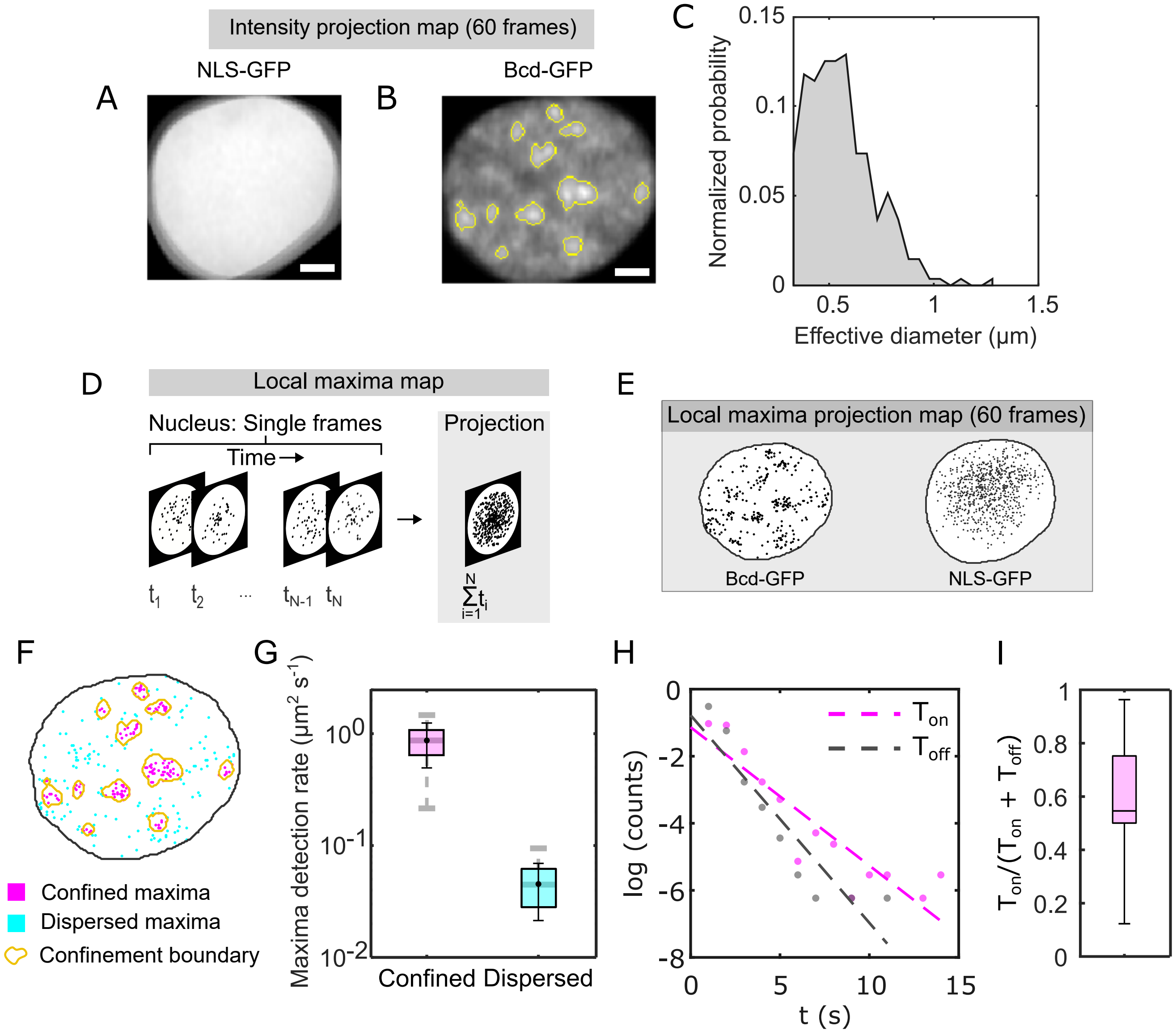

FIG. S4. **Frequency of cluster formation and lifetime of clusters.** (A, B) Time-projected images of a cross-section of a single nucleus expressing NLS-GFP (A) or Bcd-GFP (B). Movies are composed of 60 video frames, two per second. The nuclear pixels are segmented, and the centroid of the binary nuclear mask is used to register the nucleus so that the centroids across all frames are aligned. These registered nuclei from each frame are then projected onto a single frame to obtain the images seen in A and B. The uniformity of the nuclear pixels in A image suggests that any heterogeneity in individual frames is random and thus averaged out over time. In contrast, the B image shows distinct subnuclear accumulations. These accumulations indicate that local intensity maxima appear over multiple frames in those regions. These accumulations are segmented (Materials and Methods), and their boundaries are shown as yellow line overlays. These boundaries represent confinement areas where local maxima frequently appear in the movies. The scale bar is 1 μm. (C) To calculate the effective sizes of the confinement areas marked in yellow in B, the area of each detected area in pixels is calculated. An effective diameter of each area, as an approximated circle is calculated. A histogram of the respective diameters is shown in C, with $mean \pm s.d.$ of $0.53 \pm 0.16 \mu$m. (D) Schematic showing a map of local fluorescence intensity maxima inside a nucleus (left). The local maxima maps are extracted from individual frames of ~ 30 s long videos (60 frames) of nuclear cross sections (1μm thick). All maps from a given video are projected onto a single frame to form the local maxima map (right). See also Materials and Methods. (E) Representative local maxima maps for a Bcd-GFP nucleus (left) and an NLS-GFP nucleus (right) extracted from nuclei imaged for 30 seconds (60 video frames). [Captions continued on the next page]

FIG. S4. (F) The local intensity map of Bcd-GFP shown in E is repeated here. Utilizing the confinement area boundaries (in yellow), we can segregate the maxima into either confined (magenta) or dispersed (cyan) maxima. The magenta maxima occur with higher spatial density than the cyan ones. This is an indication that the confined maxima are more persistent and are candidates for clusters. (G) This disparity in spatial density of maxima is quantified here. Box plots show the rate of detection of confined (magenta) and dispersed (cyan) maxima in the projection maps (44 nuclei, 12 embryos). Boxes extend from the 25th to the 75th percentile, while the horizontal divider marks the median. Mean and standard deviations are overlaid in black (0.87 ± 0.37 and 0.04 ± 0.02 for confined and dispersed maxima respectively). These represent the spatial density of maxima of the full video projection. The spatial density of maxima per unit is obtained by dividing by the total time of the video (30 seconds), with unit $\mu m^2 s^{-1}$. The maxima in cyan could be either noise or represent highly transient or mobile clusters. The high density of maxima in magenta indicates that a cluster is detected in multiple video frames within the yellow confines. (H) In this figure, we estimate the time intervals for which confined maxima are detected (T_{on}), as well as the time interval between two successive maxima detection (T_{off}). These can help understand the persistence of a confined maximum, and thereby a cluster. The magenta data shows a scatter plot of the natural logarithm of the probability of T_{on} obtained by binning the T_{on} data into equidistant bins. An exponential fit to the data (magenta broken line) gives the time constant associated with T_{on}, $\tau_{on} = 2.4 \pm 0.3$ s. This time constant, τ_{on} serves as an estimate for the persistence time of a cluster. However, another important metric is the time constant associated with T_{off}. The probability of T_{off} split into equidistant bins is shown in the grey scatter plot. An exponential fit (grey, broken line) gives the time constant $\tau_{off} = 1.6 \pm 0.3$ s. Thus, clustering can be thought to occur at a high frequency, and this should be reflected in the fraction of time for which a cluster persists. (I) The fraction of time for which a cluster persists is given by $f_{on} = \frac{T_{on}}{T_{on}+T_{off}}$. This quantity is shown as a box plot here. Boxes extend from the 25th to the 75th percentile, while the horizontal divider marks the median. The whiskers denote the 5th and the 95th percentile of the data. The mean f_{on} was 0.58, with a total imaging time for each nucleus being 30 s. The time limit of 30 s arises out of chromatin motion which causes DNA-bound spots to drift out of the plane of imaging after that time. To summarize, the data suggests that an average cluster forms every ~ 1.5 seconds and lasts for ~ 2.5 seconds, providing both the frequency and lifetimes of clusters. This data represents nuclei located about 30 % of the length from the anterior pole of the embryo.

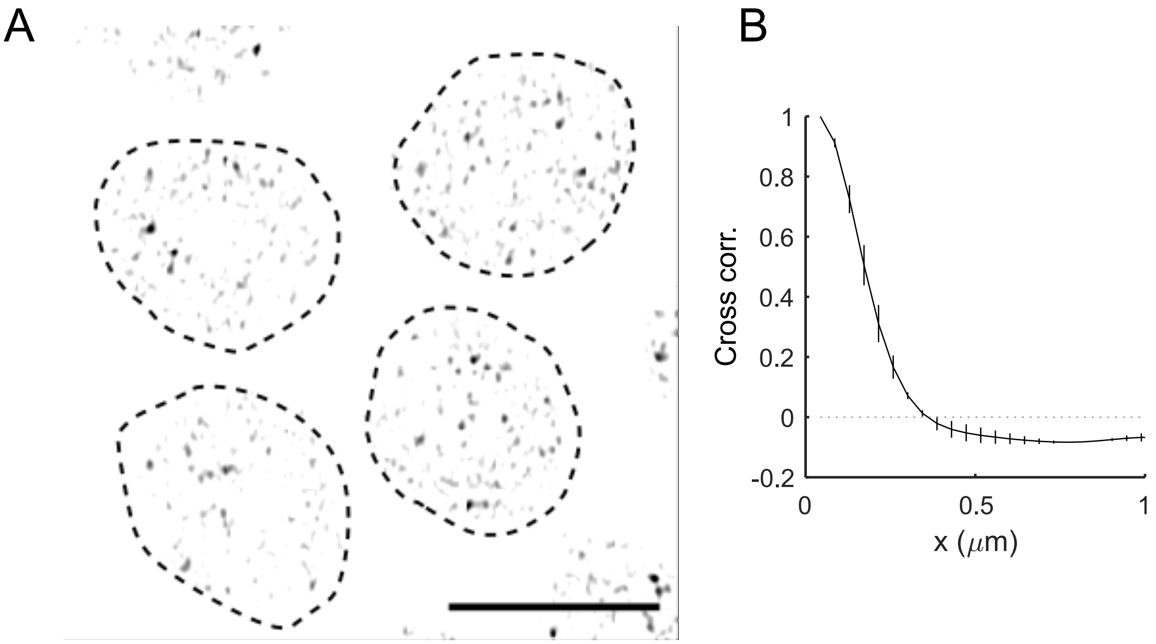

FIG. S5. **Bcd-GFP clusters in fixed embryos.** The goal of embryo fixation was to visualize Bcd clusters by immobilizing them in space. Bcd clusters form foci that are near the diffraction limit with a low signal-to-noise ratio, making it essential to avoid artifacts typically introduced by antibody staining techniques. Therefore, we utilize the fluorescence of the monomeric eGFP tag of Bcd-GFP fusion molecules to visualize the clusters after fixation. This presented two challenges: 1) preserving the fluorescence of GFP after fixation, and 2) preserving the clusters themselves. We addressed both issues by exclusively using freshly dissolved methanol-free formaldehyde (Thermo Scientific Pierce) at a final concentration of 4 % for embryo fixation. Throughout fixation and handling, we ensured that the embryos' exposure to organic solvents such as heptane, methanol, or ethanol was minimal. With these modifications to a standard protocol [52], fixation and visualization of Bcd clusters in the embryos were achieved. (A) All images and analyses shown in this manuscript are from live imaging data. However, we ensured that the clusters were also identifiable after the fixation of the embryos. The image here shows the cross-section of nuclei expressing Bcd-GFP fixed using Formaldehyde (Materials and Methods). The dashed lines are guides to the eye for the nuclear boundaries. The scale bar is 5 μm. (B) Figure shows pixel correlation function (mean±s.d., n = 15 nuclei) on the nuclear pixels of a fixed Bcd-GFP embryo. Exponential fit to the plot gives a correlation length of 0.24 ± 0.01 μm, which is similar to the correlation length in live embryos.

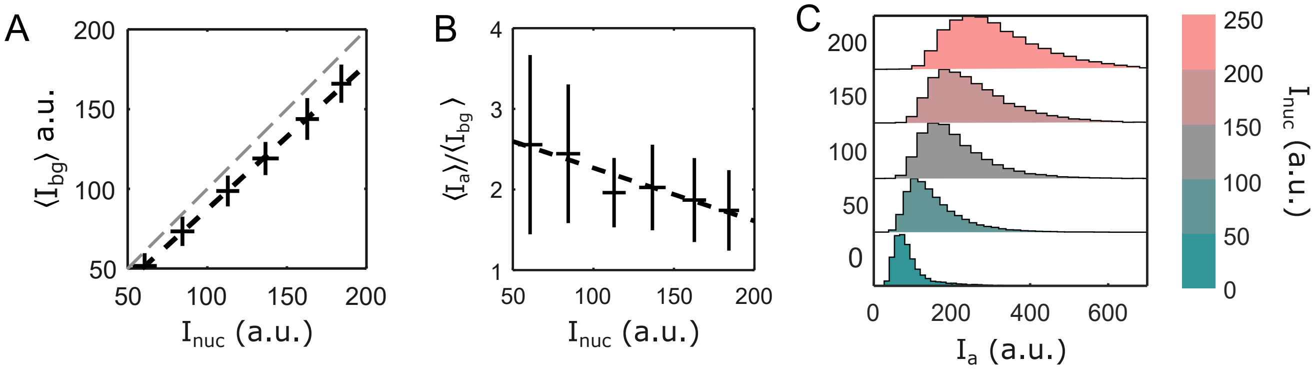

FIG. S6. **Molecular enrichment within clusters.** (A) Cluster background intensity, I_{bg}, as a function of average nuclear Bcd-GFP intensity (I_{nuc}). I_{bg} is obtained from cluster fitting (Materials and Methods) and represents the intensity of Bcd-GFP at the edge of the detected clusters. Data shows $mean \pm s.d.$, fitted with a linear function (black dashed line $R^2 = 0.95$). The values of I_{bg} are consistently lower than I_{nuc} (grey dashed line representing $y = x$). This is expected since I_{nuc} encompasses both I_{bg} and the cluster amplitude I_a. (B) The nuclear average of the ratio of the cluster amplitude and the respective cluster background intensity (I_a/I_{bg}), plotted as a function of the average nuclear intensity I_{nuc}. This ratio measures the relative enrichment of Bcd molecules in clusters. It is relatively higher for lower I_{nuc}. (C) Histograms showing the distribution of cluster amplitudes, I_a for different average nuclear intensity bins, I_{nuc}. The color bar represents ranges of I_{nuc}.

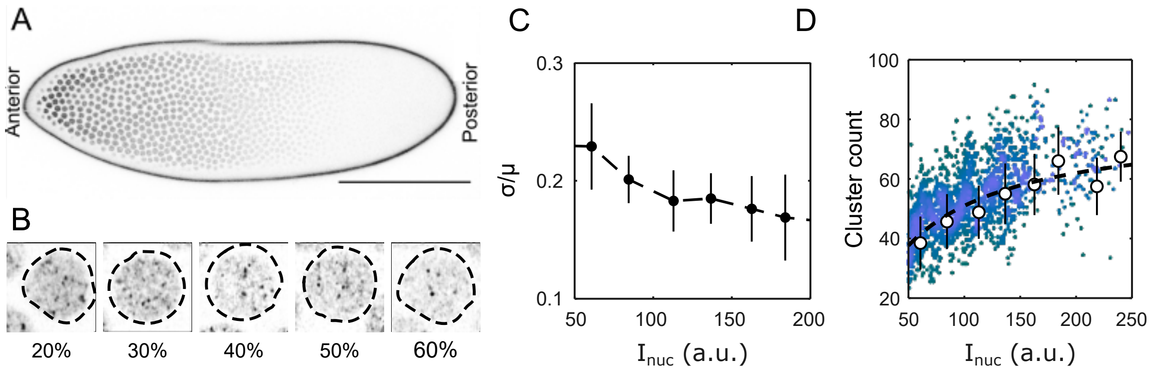

FIG. S7. **Cluster count.** (A) Cross-section of a *Drosophila* embryo in nuclear cycle 14, expressing Bcd-GFP. The focal plane was set to roughly halfway between the embryo bottom and the midsaggital plane. This image, acquired using a traditional confocal setting, has a resolution much lower than the *Airyscan* images. The anterior and posterior ends of the embryo are marked. The higher GFP intensities in the anterior nuclei (darker nuclei) indicate a higher concentration of Bcd. The scale bar is $100\,\mu$m. (B) Representative images showing cross-sections of individual nuclei expressing Bcd-GFP, imaged using the *Airyscan* mode as a function of nuclear position (anterior to posterior from left to right in percentage of embryo length). The imaging settings are the same as those used for detecting and analyzing 3D clusters. Images are $8 \times 8\,\mu$m in size. (C) Coefficient of variation (CV, σ/μ) of the cluster count per nucleus as a function of nuclear Bcd-GFP intensity (I_{nuc}). Pooled data from 14 embryos, 2027 nuclei were discretized into 10 I_{nuc} bins. Mean and standard deviation were calculated for each bin using bootstrap sampling. With an average CV less than 20 %, the cluster count displays remarkable reproducibility across embryos, comparable to that of the nuclear Bcd-GFP intensity (I_{nuc}) [35, 49]. (D) Cluster count per nucleus as a function of nuclear Bcd-GFP intensity (I_{nuc}). Despite the high reproducibility in C, the cluster count correlates poorly with I_{nuc}, as also shown in Fig. 2. Here we employ a simulation to understand the dependence of cluster counts on I_{nuc}. The scatter plot shows the number of clusters detected per nucleus. In contrast, the error bars represent the same data discretized into 10 bins and the mean and the standard deviation of the data within these bins were calculated by bootstrap sampling. The dashed black line illustrates the simulated dependence of the cluster counts on I_{nuc}. The simulation uses an empirical relation between the cluster count and I_{nuc}, with a model that hinges on the probability that a cluster is bound to its seeding site, denoted as $p_{on} = t_{on}/(t_{on} + t_{off})$. While t_{on} represents the time for which the cluster is "bound", and hence detectable, t_{off} is the time for which the cluster is "unbound" and thus not detectable. To calculate these, three simple assumptions were made: 1. The total number of seeding sites in a nucleus is constant, denoted by N. 2. The "bound" time, t_{on} for a cluster i is determined solely by the properties of its seeding site, $n(i)$. 3. The "unbound" time, t_{off} depends on diffusion parameters and is inversely proportional to the nuclear concentration c, which correlates with I_{nuc}. Therefore, the sum $\sum_{i=1}^{N} p_{on}(i,c)$ provides the total number of clusters detected per nucleus. We used $N = 80$, which represents the maximum number of seeding sites within a nucleus in an embryo, with $n(i)$ being randomly generated.

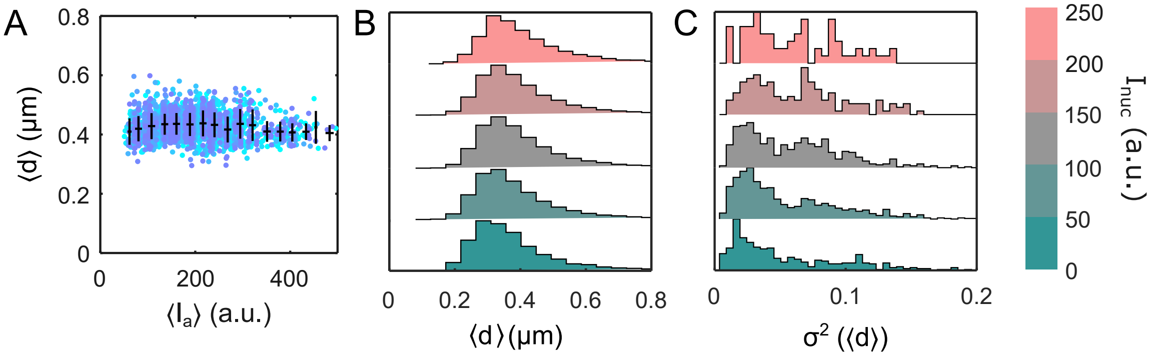

FIG. S8. **Cluster size.** (A) In Fig. 2F, we showed that the cluster diameter d is uncorrelated with the nuclear concentration of Bcd-GFP, I_{nuc}. Panel A shows that d is also uncorrelated with the cluster's Bcd-GFP concentration, I_a. (B) Cluster diameter distributions for different I_{nuc} bins. The range of I_{nuc} per histogram is indicated by the color bar on the right. Histograms are invariant of I_{nuc} range and thus d is independent of the nuclear concentration, I_{nuc}. (C) Histograms of cluster size variances $\sigma^2(d)$, binned and sorted according to the same I_{nuc} ranges as in B (color as in B).

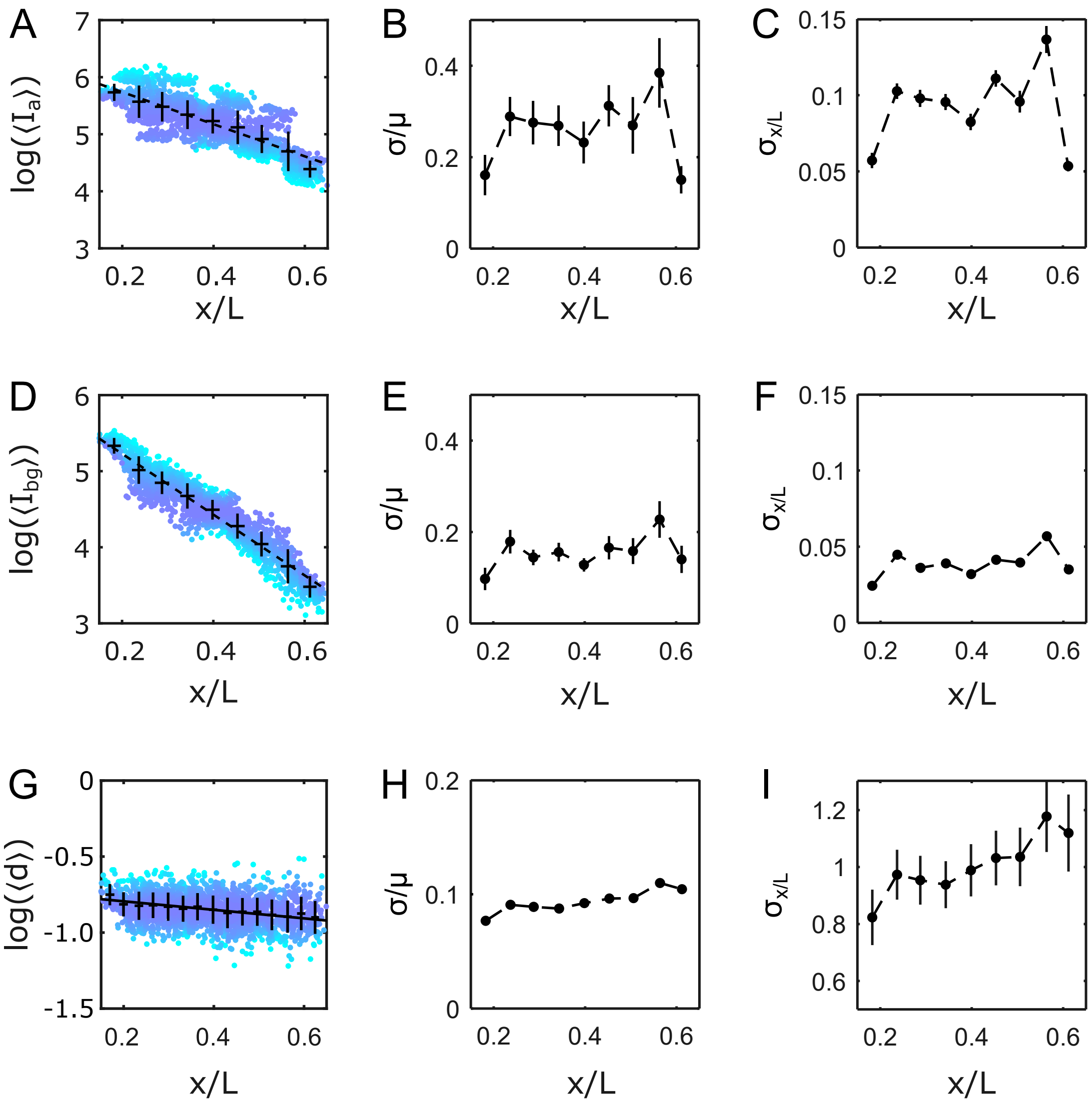

FIG. S9. **Precision of nuclear position determination from cluster parameters.** (A) Scatterplot of the nuclear average of Bcd-GFP cluster amplitudes ($\langle I_a \rangle$, log units) as a function of fractional egg length x/L. Data points are for individual nuclei (14 embryos, 2027 nuclei). Data is discretized into equidistant bins, and mean and s.d. (error bars) are calculated via bootstrap sampling from all nuclei within each x/L bin. The exponential decay constant extracted from a linear fit (dashed line) is $\lambda = 0.32 \pm 0.03$ EL. (B) Coefficient of Variation (CV) of $\langle I_a \rangle$ as a function of x/L in the same bins as in A. The average CV is $\sim 30\,\%$, higher than that of I_{nuc} ($\sim 15\,\%$, see Fig. 3B). (C) Figure shows the precision of nuclear position $\sigma(x)$ determined using $\langle I_a \rangle$. Utilizing $\frac{\langle I_a \rangle}{dx}$, from A and $\delta\langle I_a \rangle$ from B, we can derive $\sigma(x)$ as $\sigma(x) = \delta\langle I_a \rangle |\frac{\langle I_a \rangle}{dx}|^{-1}$. The average precision is $\sim 10\,\%$, which is equivalent to ~ 4 nuclear width. Thus $\langle I_a \rangle$ is less precise in the determination of the nuclear position than $\langle I_c \rangle$ ($\sim 5\,\%$, see Fig. 3C). (D) Scatterplot of the nuclear average of Bcd-GFP cluster background intensity ($\langle I_{bg} \rangle$, log units) as a function of x/L. Data is discretized into equidistant bins, and mean and s.d. (error bars) are calculated via bootstrap sampling from all nuclei within each x/L bin. The exponential decay constant extracted from linear fit (dashed line) is $\lambda = 0.25 \pm 0.02$ EL, which is similar to the gradient of I_{nuc}. (E) The CV of $\langle I_{bg} \rangle$ expressed as a function of x/L. The average CV is $< 20\%$. Thus $\langle I_{bg} \rangle$ has lesser variability than $\langle I_a \rangle$ (B). (F) The precision of nuclear position determination using the average $\langle I_{bg} \rangle$ is shown here. The precision is calculated as in C and is given by, $\sigma(x) \sim 4\,\%$, which is equivalent to ~ 1.5 nuclear width, similar to the precision in I_{nuc}. This is because $\langle I_{bg} \rangle$ represents the diffusing Bcd molecules which constitute $> 90\,\%$ of the molecules in the nucleus. Here $\sigma(x) = \delta\langle I_{bg} \rangle(x) |\frac{\langle I_{bg} \rangle(x)}{dx}|^{-1}$, where $\frac{\langle I_{bg} \rangle(x)}{dx}$ is obtained from D and $\delta\langle I_{bg} \rangle(x)$ is obtained from E. (G) Scatterplot of the nuclear average of Bcd-GFP cluster size, $\langle d \rangle$ (log units) as a function of x/L. Data is discretized into equidistant bins, and mean and s.d. (error bars) are calculated via bootstrap sampling from all nuclei within each x/L bin. The exponential decay constant extracted from linear fit (dashed line) is $\lambda = 10.7 \pm 0.9$ EL. (H) The CV of $\langle d \rangle$ is expressed as a function of x/L. The average CV is $\sim 10\,\%$. Thus the average size, $\langle d \rangle$ has the lowest variability among all cluster parameters. (I) The precision of nuclear position determination using the average $\langle d \rangle$. The precision is $\sigma(x) = \sim 100\,\%$, which is equivalent to ~ 1 embryo length. This shows that $\langle d \rangle$ can not sense nuclear position. Even though $\langle d \rangle$ is a highly reproducible quantity among embryos, it fails to carry positional information due to its weak dependence on position (x/L). Calculation of $\sigma(x)$ follows the same logic as in C and F, giving $\sigma(x) = \delta\langle d \rangle(x) |\frac{\langle d \rangle(x)}{dx}|^{-1}$, where $\frac{\langle d \rangle(x)}{dx}$ is obtained from G and $\delta\langle d \rangle(x)$ is obtained from H.

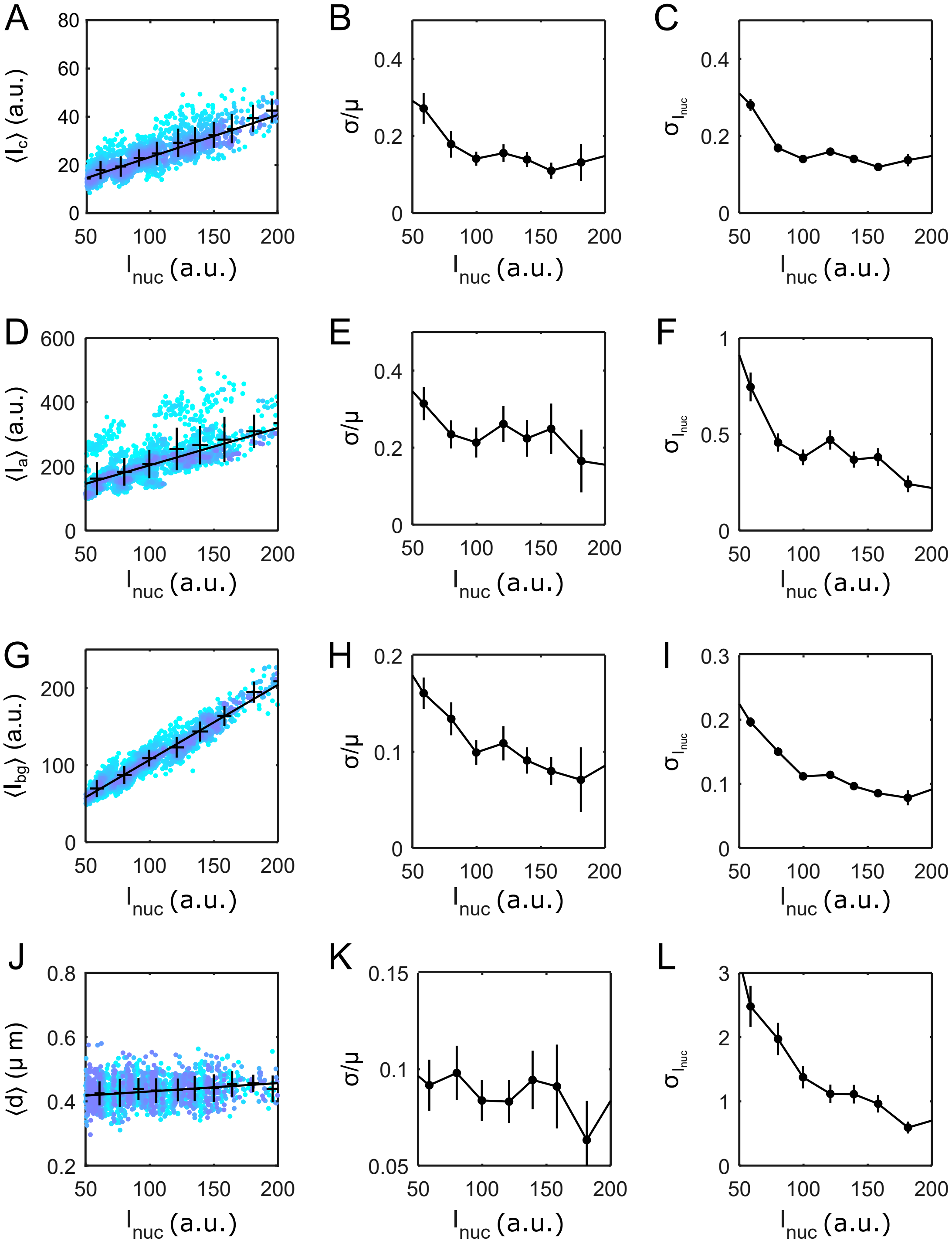

FIG. S10. **Precision of nuclear concentration determination from cluster parameters.** (A) Scatterplot of the nuclear average of Bcd-GFP cluster total intensity, $\langle I_c \rangle$ expressed as a function of nuclear Bcd-GFP intensity I_{nuc}. A cluster's total intensity is a measure of the total number of Bcd-GFP molecules within a cluster. Data points are for individual nuclei (14 embryos, 2027 nuclei). Data is discretized into equidistant bins, and mean and s.d. (error bars) are calculated via bootstrap sampling from all nuclei within each x/L bin. The slope from the linear fit (solid line) is used to extract the precision of I_{nuc} determination using $\langle I_c \rangle$. (B) Coefficient of Variation (CV) of $\langle I_c \rangle$ expressed as a function of I_{nuc}. The average variability is $\sim 15\,\%$. [Caption is continued on the next page.]

FIG. S10. (C) The precision of estimating I_{nuc} utilizing $\langle I_c \rangle$ is given by $\sigma_{I_{nuc}} = \delta\langle I_c\rangle(c)|\frac{\langle I_c\rangle(c)}{dc}|^{-1}$, by combining the slope found from the linear fit in A ($\frac{\langle I_c\rangle}{dc}$) and the $\delta\langle I_c\rangle$ in B. Here, c is the concentration of Bcd-GFP in a cell. The average precision of I_{nuc} determination using the average $\langle I_c\rangle$ is 0.23 ± 0.07. Precision is relatively higher for the anterior nuclei at $\sim 15\%$. Since the CV of I_{nuc} is $\sim 15\,\%$, $\langle I_c\rangle$ can be used to determine Bcd's cellular concentration c with a precision of ~ 1 cell. (D) Scatterplot of the nuclear average of Bcd-GFP cluster amplitude, $\langle I_a\rangle$ expressed as a function of nuclear Bcd-GFP intensity I_{nuc}. Data points are for individual nuclei (14 embryos, 2027 nuclei). Data is discretized into equidistant bins, and mean and s.d. (error bars) are calculated via bootstrap sampling from all nuclei within each x/L bin. The slope from the linear fit (solid line) is used to extract the precision of I_{nuc} determination using $\langle I_a\rangle$. The slope of the linear fit is $\frac{\langle I_a\rangle(c)}{dc} = 1.19 \pm 0.10$ EL. (E) The CV of $\langle I_a\rangle$ expressed as a function of I_{nuc}. The average CV is $\sim 25\,\%$ is higher than that of $\langle I_c\rangle$. (F) The precision of estimating I_{nuc} utilizing $\langle I_a\rangle$ is given by $\sigma_{I_{nuc}} = \delta\langle I_a\rangle(c)|\frac{\langle I_a\rangle(c)}{dc}|^{-1}$, by combining the slope found from the linear fit in D ($\frac{\langle I_a\rangle(c)}{dc}$) and the $\delta\langle I_a\rangle(c)$ in E. Here, c is the concentration of Bcd-GFP in a cell. The average precision of I_{nuc} determination using the average $\langle I_a\rangle$ is 0.43 ± 0.20, equivalent to $\sim 40\%$ error. Thus, $\langle I_a\rangle$ is less precise in the determination of the I_{nuc} than $\langle I_c\rangle$, with a precision of ~ 3 cells. (G) Scatterplot of the nuclear average of Bcd-GFP cluster background intensity, $\langle I_{bg}\rangle$ expressed as a function of nuclear Bcd-GFP intensity I_{nuc}. Data points are for individual nuclei (14 embryos, 2027 nuclei). Data is discretized into equidistant bins, and mean and s.d. (error bars) are calculated via bootstrap sampling from all nuclei within each x/L bin. The slope from the linear fit (solid line) is used to extract the precision of I_{nuc} determination using $\langle I_{bg}\rangle$. The slope of the linear fit is $\frac{\langle I_{bg}\rangle(c)}{dc} = 0.99 \pm 0.02$ EL. (H) The CV of $\langle I_{bg}\rangle$ is expressed as a function of I_{nuc}. The average CV is $\sim 10\,\%$, making it a highly reproducible quantity, similar to I_{nuc} (Fig. 3B). (I) The precision of estimating I_{nuc} utilizing $\langle I_{bg}\rangle$ is given by $\sigma_{I_{nuc}} = \delta\langle I_{bg}\rangle(c)|\frac{\langle I_{bg}\rangle(c)}{dc}|^{-1}$, by combining the slope found from the linear fit in G ($\frac{\langle I_{bg}\rangle(c)}{dc}$) and the $\delta\langle I_{bg}\rangle(c)$ in H. Here, c is the concentration of Bcd-GFP in a cell. The average precision of I_{nuc} determination using $\langle I_{bg}\rangle$ is $\sim 10\,\%$ in the anterior, making the precision of concentration determination using $\langle I_{bg}\rangle$ as precise as a single cell. This is not surprising since $\langle I_{bg}\rangle$ values represent the freely diffusing Bcd molecules, which constitute $> 90\,\%$ Bcd molecules in the nucleus. (J) Scatterplot of the nuclear average of Bcd-GFP cluster size, $\langle d\rangle$ expressed as a function of nuclear Bcd-GFP intensity I_{nuc}. Data points are for individual nuclei (14 embryos, 2027 nuclei). Data is discretized into equidistant bins, and mean and s.d. (error bars) are calculated via bootstrap sampling from all nuclei within each x/L bin. The slope from the linear fit (solid line) is used to extract the precision of I_{nuc} determination using $\langle d\rangle$. (K) The CV of $\langle d\rangle$ is expressed as a function of I_{nuc}. The average variability is $< 10\,\%$. (L) The precision of estimating I_{nuc} utilizing $\langle d\rangle$ is given by $\sigma_{I_{nuc}} = \delta\langle d\rangle(c)|\frac{\langle d\rangle(c)}{dc}|^{-1}$, by combining the slope found from the linear fit in J ($\frac{\langle d\rangle(c)}{dc}$) and the $\delta\langle d\rangle(c)$ in K. Here, c is the concentration of Bcd-GFP in a cell. The average precision of I_{nuc} determination using the average $\langle d\rangle$ is $> 100\%$, making cluster size an extremely imprecise metric for estimating I_{nuc}.

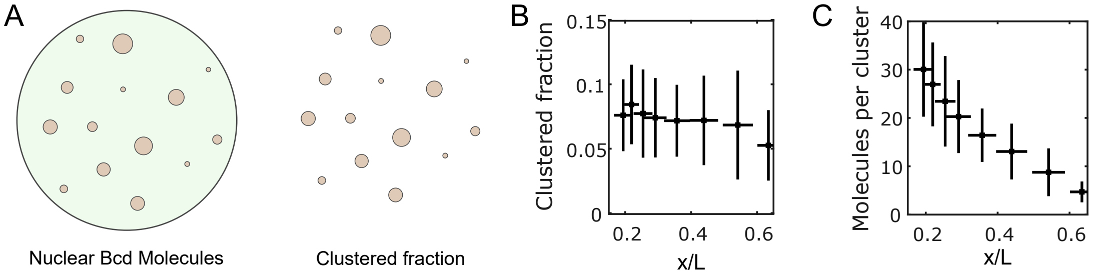

FIG. S11. **Estimation of the number of molecules per cluster.** (A) A cartoon depicting the broad distribution of Bcd molecule assemblies in the nucleus. While green depicts the freely diffusing Bcd molecules in the background, brown circles (also separately shown on the right) represent the clustered fraction. (B) Figure shows the fraction of Bcd molecules in the clustered fraction per nucleus as a function of the nuclear position in the embryo (error bars represent mean ± s.d. each calculated via bootstrap sampling). The cluster volume and intensity were multiplied and summed over all clusters in a given nucleus; the resulting sum was divided by the product of the nuclear intensity and the nuclear volume to obtain the fraction of Bcd molecules. (C) The average Bcd molecule count per cluster (mean ± s.d., each calculated via bootstrap sampling) plotted as a function of nuclear position in the embryo. The count was obtained by first calculating the absolute number of Bcd molecules in a nucleus utilizing the count obtained in [21], and then multiplying with the fraction in B (see Materials and Methods). The average number of molecules drops from about 30 at the anterior to 5 at 60% of the embryo. Note that the experimental detection limit is at ~ 5 molecules per cluster.

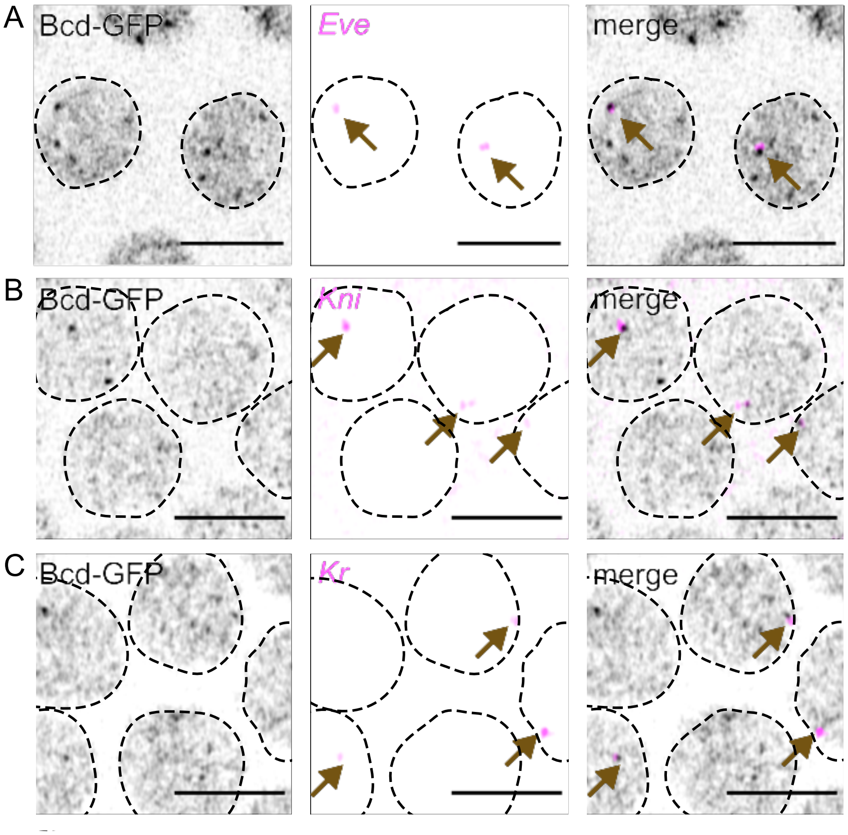

FIG. S12. **Colocalization of Bcd cluster with nascent transcription sites.** Representative raw images of nuclei expressing Bcd-GFP (left panels). The middle panels show the same nuclei as on the left with sites of nascent transcription of *eve* (A), *kni* (B), and *Kr* (C). The right panels show the overlay of the left and middle images. Arrows indicate transcription sites.

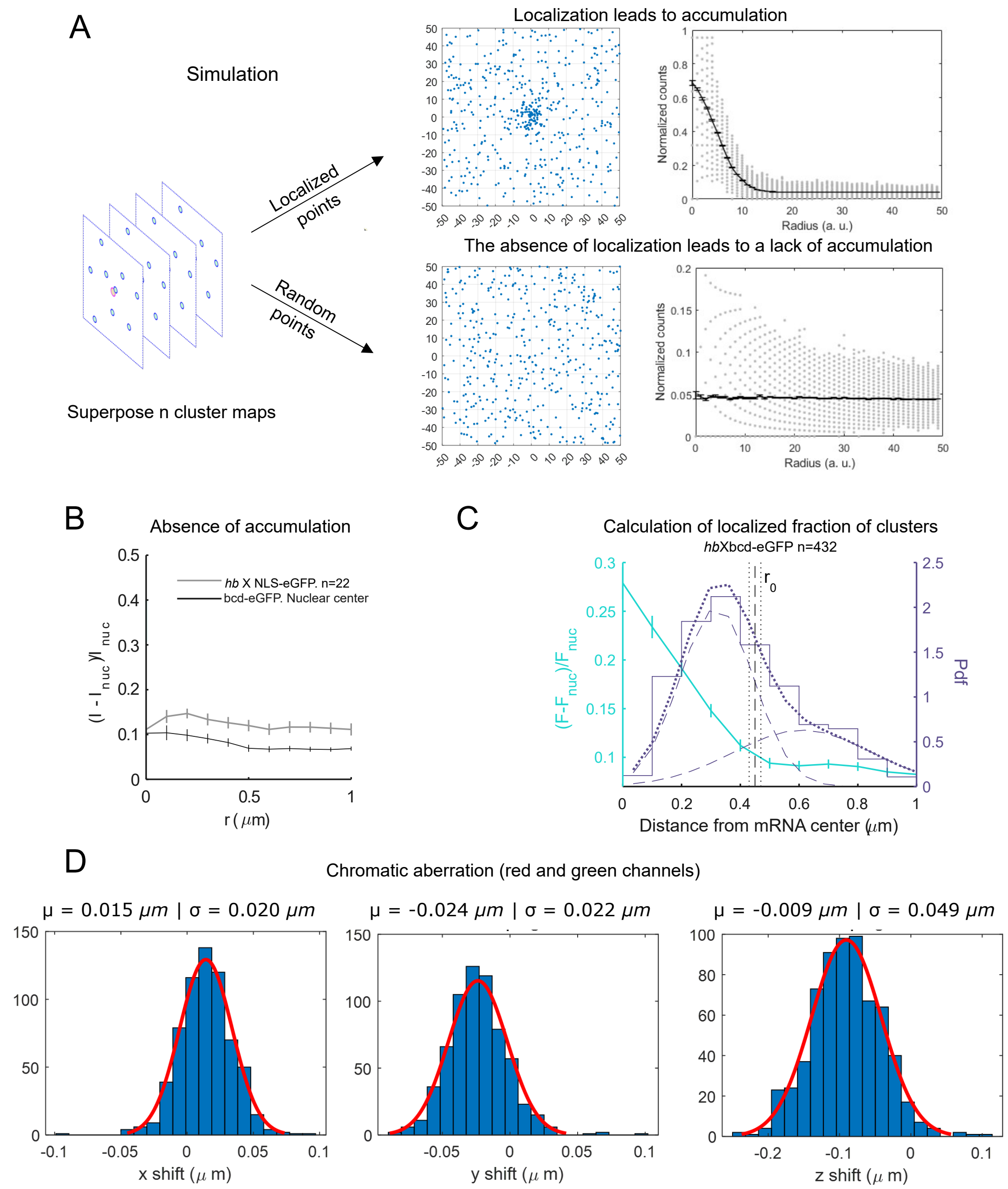

FIG. S13. **Bcd cluster coupling with site of nascent transcription.** [For caption see the next page]

FIG. S13. **Bcd cluster coupling with site of nascent transcription.** (A) Computer simulation of transcription factor diffusion in a space with or without a nascent transcription hotspot at its center. (Left) Cartoons representing a nascent transcription hotspot (pink) and TF molecules (blue). (Right, Top) Diffusing TF molecules (blue dots) at a given time are distributed as a Poisson point process across the simulation window. With a seeding site at the origin (emulating a nascent transcription hotspot), TF molecules preferentially accumulate there in a Gaussian-distributed fashion. This accumulation (or lack thereof) is better characterized by calculating the radial density of TF molecules, as shown on the left. The top plot shows the Gaussian distribution describing the accumulation of the TF molecules, the FWHM of which effectively gives the radius of the spread of the molecules around the seeding site. Molecular clusters appearing within a radial limit given by $r_0 = 2 \times FWHM$ are considered to be coupled to the seeding site at the origin in some capacity. This is elucidated in C. (Right, Bottom) The absence of a seeding site at the origin leads to a random distribution of molecules, yielding a flat profile of the radial distribution of TF molecule density (RIGHT). Examples of this can be seen in B. (B) Here we show examples of the lack of accumulation of molecules, complementary to Fig. 4C, showing accumulation of Bcd around target genes. For this, we imaged NLS-GFP expressing nuclei, in which a transcription site of *Hb* gene was also labeled with MCD-mRuby3. Since *Hb* is not a target gene of NLS, no accumulation was observed (gray). We could also replicate this lack of accumulation by imaging Bcd-GFP nuclei taking radial intensity profile around the nuclear center, which can be assumed to be a Bcd agnostic site (black). (C) This plot tests the hypothesis that the molecular clusters coupled to a seeding site are preferentially found within $r_0 = 2 \times FWHM$ of the radial accumulation of molecules around the seeding site. For this, we plot the 2D radial intensity of Bcd-GFP around a *Hb* mRNA hotspot (Cyan, mean $\pm$ s.d.). The cyan fit gives a double Gaussian fit. The vertical lines show mean $\pm$ standard error in the calculation of $r_0 = 2 \times FWHM$ from the first of the two fitting Gaussians. In the same plot, the histogram of the distances of the clusters nearest to the *Hb* mRNA hotspot is plotted. The nearest clusters can either be coupled (when seeded at the *hb* site) or uncoupled, in which case the second-nearest cluster would be registered as the nearest one. To account for this, this histogram is fitted with a double Gaussian, one representing the coupled nearest cluster and the other the uncoupled nearest cluster. The Gaussian kernels along with the double Gaussian fit are shown in broken blue lines. The position of the intersection of the two Gaussian kernels gives the threshold distance separating the coupled from the uncoupled nearest clusters. This intersection is only $\sim 50\, nm$ from r_0, obtained above, which is comparable to the dimension of a single pixel (43 nm). This warrants the use of the FWHM obtained from the radial intensity plots to find the coupling fraction of clusters in Fig. 4H and Fig. S14B. (D) Histograms of shifts in the intensity-weighted centers of polystyrene beads measured in two color channels, red and green. A Gaussian fit to each histogram gives the corresponding σ which serves as a measure of chromatic aberration along each image axis. All three shifts are sub-pixel.

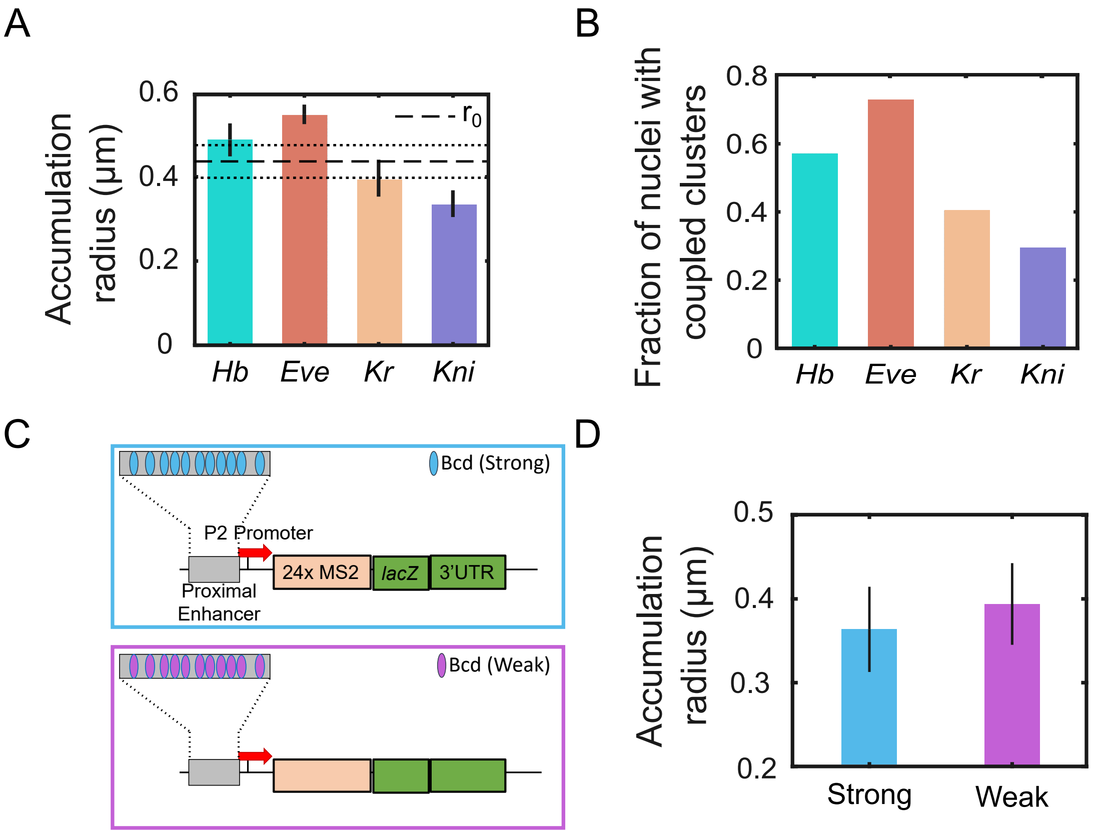

FIG. S14. **Fraction of transcription hotspot coupled with a Bcd cluster.** (A) Bars representing 2σ from the half-normal fits of Bcd-GFP accumulation (see Materials and Methods) for each target gene (indicated on the x-axis). Whiskers indicate standard errors. The horizontal line shows the average (dashed) and standard error bounds(dotted) from the entire dataset, calculated via bootstrapping. This average radius is also shown in Fig. 4C by a vertical dashed line. (B) The fraction of nuclei in which expressing target genes are associated with a coupled cluster. The corresponding genes are indicated on the x-axis. (C) Schematic shows the two constructs, one driven by an enhancer with strong Bcd binding sites, and the other with an enhancer composed of weak Bcd binding sites. (D) Bars representing 2σ from the Half normal fits of Bcd-GFP accumulation for the two constructs shown in C. Following the logic in Fig. S13 C, we see that the accumulation radii of both constructs are the same. This is likely because the coupled cluster location is the same for both constructs, as the enhancer is placed at the same distance from the promoter. However, in the case of the weak constructs fewer clusters are coupled Fig. 4H, hence the nearest cluster distance is larger (Fig. 4G) than the strong construct.

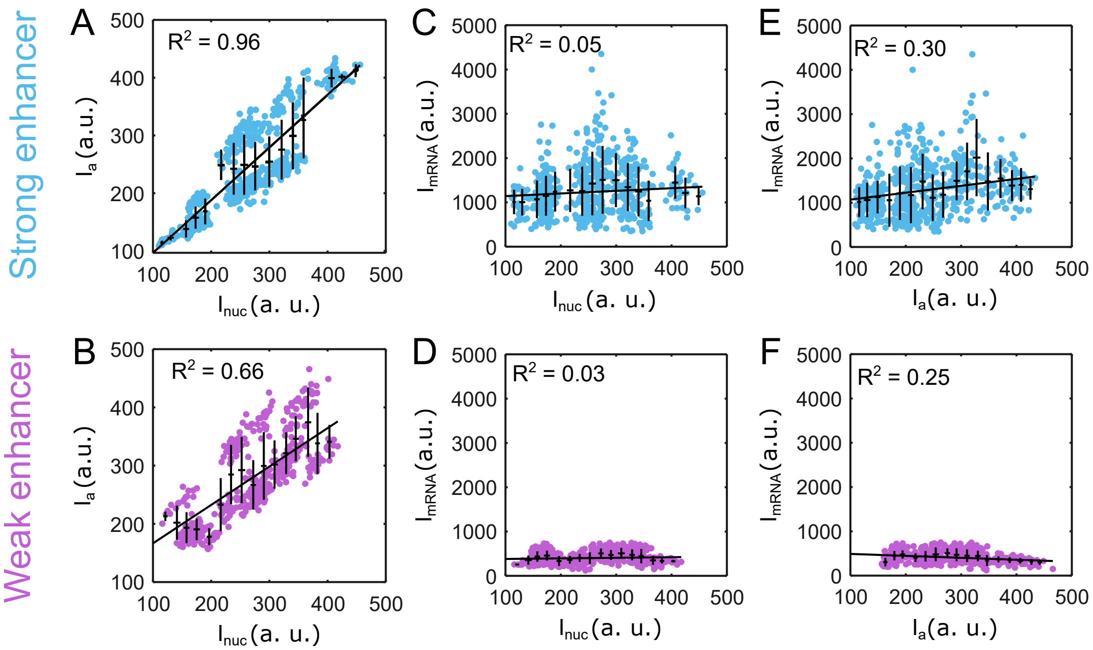

FIG. S15. **Dependence of cluster and transcription hotspot intensity on nuclear Bcd concentration for strong and weak synthetic enhancer constructs.** (A, B) Scatter plots overlaid with binned mean ± s.d. of the cluster amplitude (I_a) of the Bcd cluster nearest to the mRNA hotspot against the nuclear Bcd concentrations for strong (A, 541 nuclei, 17 embryos) and weak (B, 406 nuclei, 20 embryos) enhancers. The mean and standard deviations were each calculated via bootstrap sampling. Each plot is linearly fitted, and the corresponding R^2 value is indicated in the figure. A very high correlation (0.85, Pearson) is observed between the nearest I_a and I_{nuc} for the strong enhancer (A), while a relatively weaker correlation (0.77, Pearson) is observed for the weak enhancer. (C, D) Scatter plots overlaid with binned mean ± s.d. of the intensity of the mRNA hotspot against the nuclear Bcd concentrations for strong (C) and weak (D) enhancers. The mean and standard deviations were each calculated via bootstrap sampling. Each plot is linearly fitted, and the corresponding R^2 value is indicated in the figure. The intensity of the nascent mRNA hotspot and I_{nuc} are uncorrelated for both enhancer constructs. (E, F) Scatter plots overlaid with binned means and standard deviations of the intensity of the mRNA hotspot against the intensity of the Bcd cluster nearest to the mRNA hotspot for strong (E) and weak (F) enhancers. The mean and standard deviations were each calculated via bootstrap sampling. The mean and standard deviations were each calculated via bootstrap sampling. Each plot is linearly fitted, and the corresponding R^2 value is indicated in the figure. The correlations observed between the two quantities are weak for both enhancer constructs. This may be due to the significantly different characteristic persistence times of Bcd clusters and transcriptional bursts.

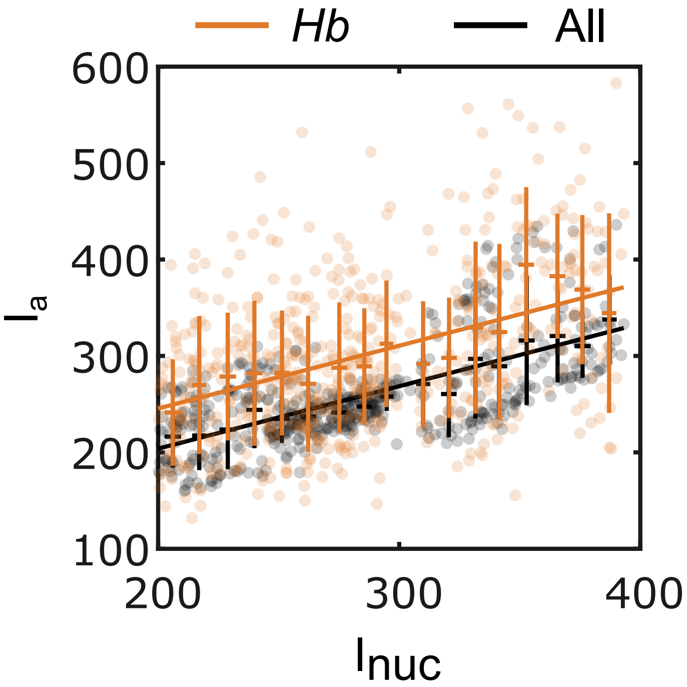

FIG. S16. **Cluster nearest to *Hb* is brighter than the average cluster.** Scatter plot showing the average Bcd intensities of clusters closest to the mRNA hotspot in a *Hb* reporter construct (orange); for comparison, average intensity of all clusters in a given nucleus in black (423 nuclei, 23 embryos). Overlaid are mean ± s.d. of I_a calculated over equal I_{nuc} bins. The linear fits, shown for data of corresponding colors (R^2 = 0.93 for the nearest Bcd cluster, R^2 = 0.71 for the average of all clusters) are guides to the eye. Thus, the Bcd-GFP clusters nearest to the *hb* locus are brighter than an average cluster in the nucleus.

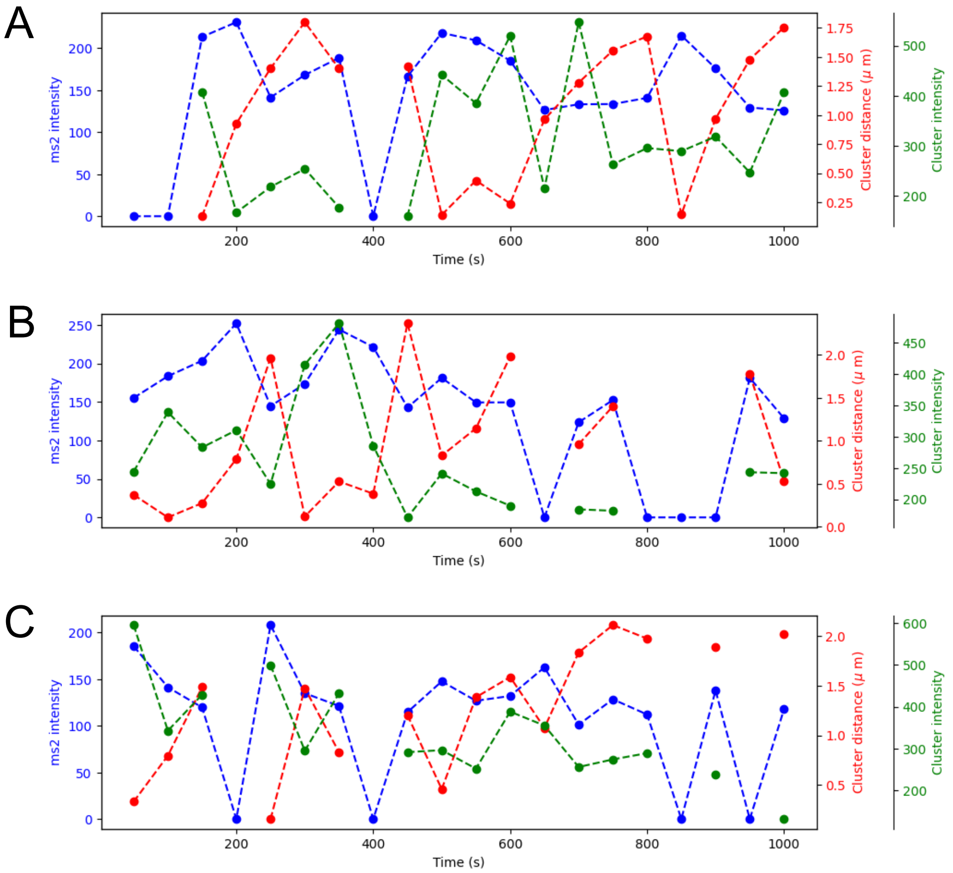

FIG. S17. **Nearest TF cluster distance and the mRNA hotspot intensity.** To understand how the nearest cluster affects gene transcriptional output, we imaged nuclei expressing Bcd-GFP and MCP-mRuby3 in 3D. The endogenous *eve* locus was tagged with MS2 stem-loops (Materials and Methods). For each video frame, we computed the intensity of the mRNA hotspot and the relative distance and intensity of the nearest TF cluster. All imaging was done at the location of the second *eve* stripe expression, during nuclear cycle 14, from 25 to 40 minutes after the end of mitosis 13. (A, B, C) Show example traces of mRNA hotspot intensity (blue), nearest Bcd cluster distance in μm (red), and nearest Bcd cluster intensity (green) for an endogenously tagged *eve* locus as a function of time. An increase in the nearest cluster distance is followed by a decrease in mRNA hotspot intensity, while no pattern is observed between the nearest cluster intensity and mRNA hotspot intensity.

II. SUPPLEMENTAL TABLES

Fly line	Source
hb BAC<MS2	Bothma *et al.*[54]
kni BAC<MS2	Bothma *et al.*[54]
eve MS2	Chen *et al.*[46]
kr MS2	El-Sherif *et al.*[55]
bnk MS2	This work
P2 (Strong) MS2	This work
P2 (Weak) MS2	This work

TABLE I. **List of MS2 stem loop fly lines.**
