# Supplementary material for: Transcription factor clusters as information transfer agents
Source: ArXiv. 2024 Mar 5:arXiv:2403.02943v1. Preprint. [Version 1] (PMC10942473)
Supplement: Supplementary file 1 [file NIHPP2403.02943V1-supplement-1.pdf]

I. SUPPLEMENTAL FIGURES

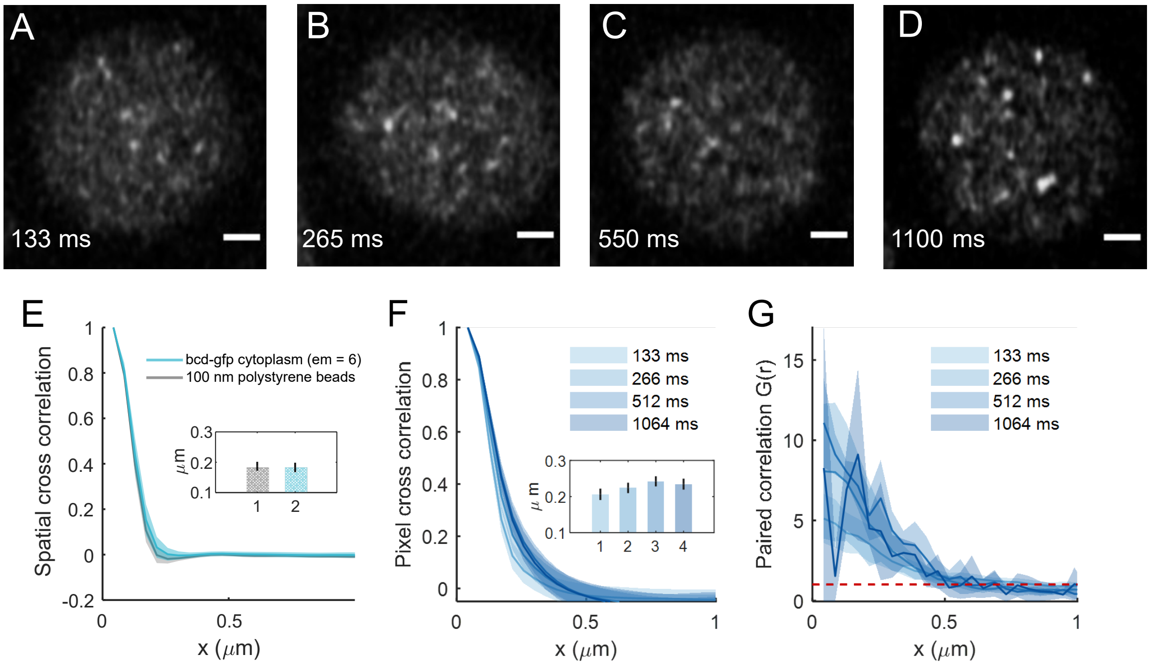

FIG. S1. **Imaging frame times and correlation lengths.** (A-D) Representative images of nuclei expressing Bcd-GFP, at different frame times (mentioned on panels). The frame time is increased by increasing the pixel dwell time of imaging. Increasing pixel dwell time blurs the more transient (or mobile) features into the background, leaving only the brighter, more stable ones in the image. The scale bars are $1\mu m$ long. (E) Pixel cross-correlation plotted for different frame times. (mean and standard error of mean are shown as error bars in the inset). The correlation lengths are statistically indifferent, indicating that motion induced blurring does not cause significant broadening of resolvable structures even at the frame time of $\sim 1\ s$ (F) Pixel correlation plot of cytoplasmic Bcd-GFP. The correlation length is $0.187 \pm 0.015\ \mu m$, a value comparable to the PSF width, indicating that Bcd in the cytoplasm is predominantly diffusive.

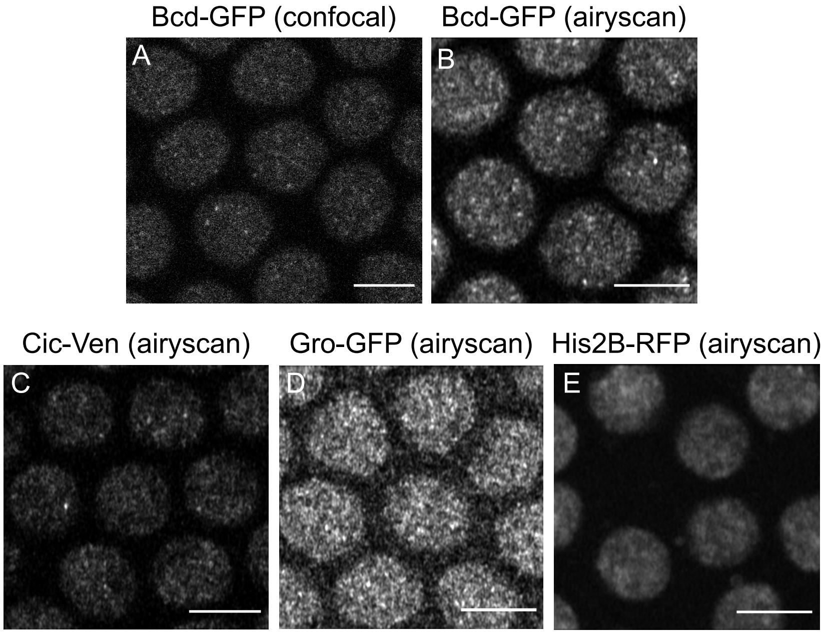

FIG. S2. **Micrographs of labeled proteins in the nuclei of *Drosophila* embryo.** (A-B) Traditional confocal (A) as well as *Airyscan* confocal images showing cross sections of Bcd-GFP expressing nuclei of live NC14 embryos. (C-E) *Airyscan* confocal images showing nuclei expressing Capicua tagged with Venus, Groucho tagged with monomeric eGFP, and Histone2B tagged with RFP in live NC14 embryos.

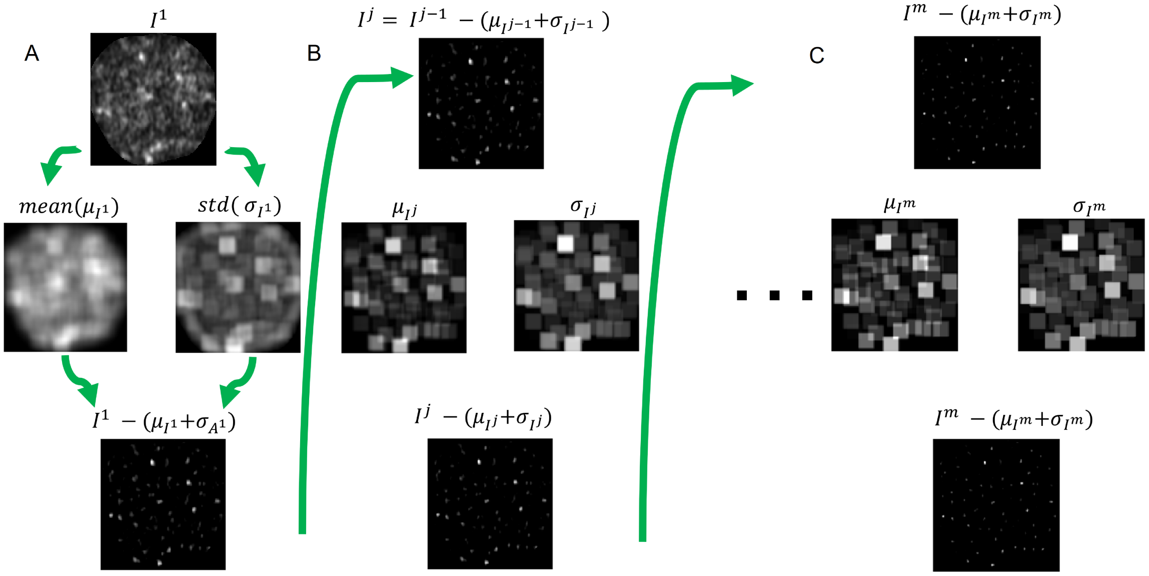

FIG. S3. **Local maxima detection technique.** (A) Image shows the raw image of a nucleus expressing Bcd-GFP (TOP). Windowed mean filtered image derived from (MIDDLE, LEFT) and windowed standard deviation filtered image derived from the top (MIDDLE, RIGHT). The image obtained after adding the images in the MIDDLE row and subtracting the result from the image at the TOP is shown in the BOTTOM. This image is the result for one layer of local filter application, and is used for the second layer of local filter application in B. (B) The image obtained in (A, BOTTOM) is recursively filtered, following the same scheme in A to obtain B (BOTTOM). This operation is repeated n times to obtain the final filtered image in C (BOTTOM).

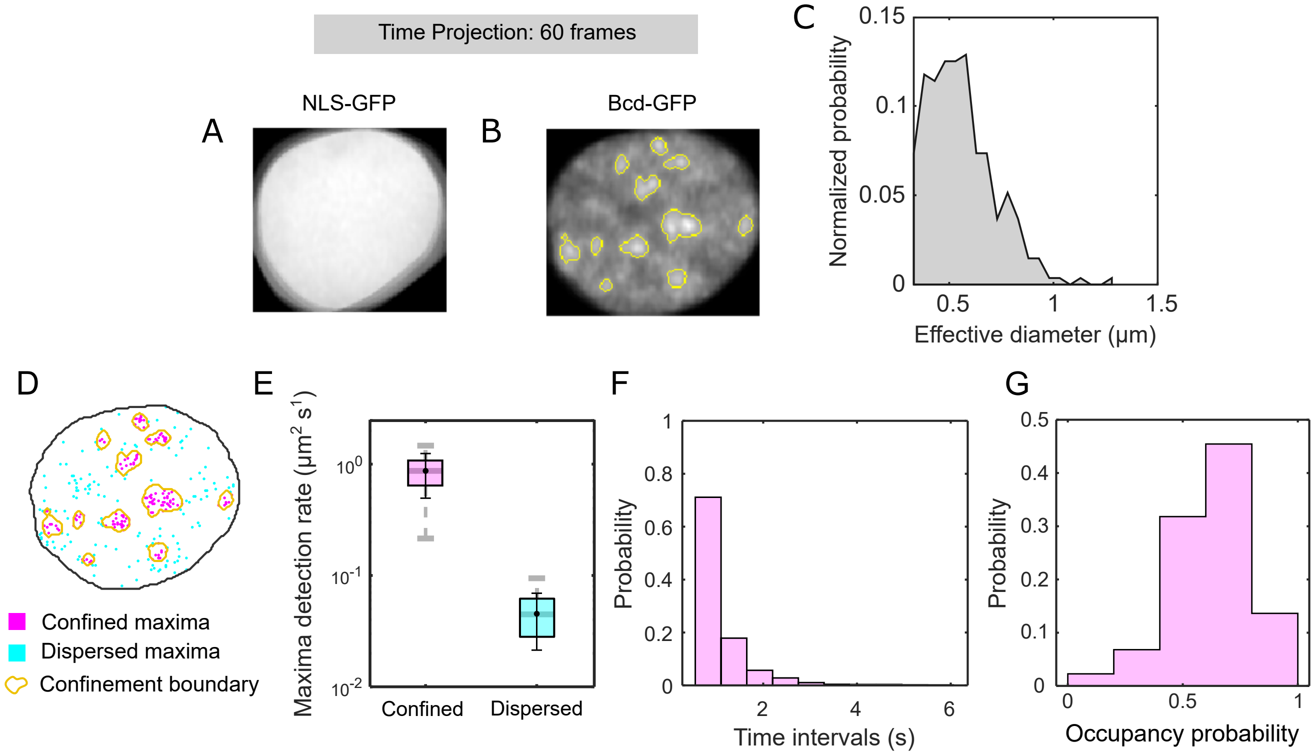

FIG. S4. **Characterization of local maxima accumulation over time.** (A, B) Each frames of movies of nuclei expressing (A) NLS-GFP and (B) Bcd-GFP (60 video frames, $\sim$ 30 seconds) are rescaled to $[0, 1]$. The nuclei are then registered and projected onto a single frame. The projection images in A and B show clear visual differences. The image in A is highly uniform, suggesting that any heterogeneity in individual frames are random and average out over time. Whereas, the image in B shows distinct subnuclear accumulations. These accumulations are segmented and the accumulation boundaries are shown as yellow line overlays. These boundaries represent confinement areas within which local maxima tend to appear in high frequency in the movies. (C) If the confinement areas in B are approximated by circles, the histogram of the respective diameters is shown in C. The $mean \pm std$ of the diameters of the confinement area is $529 \pm 164\,nm$. (D) We can now revisit the local intensity maxima map projected from all the video frames (FIG. 1E, left). Utilizing the confinement area boundaries (in yellow), we can segregate the maxima into either confined (magenta) or dispersed (cyan) maxima. Visually, the magenta maxima occur with higher spatial density than the cyan ones. (E) This disparity in density can be quantified. Box plots show the rate of detection of confined (magenta) and dispersed (cyan) maxima in the projection maps (44 nuclei, 12 embryos). Boxes extend from the 25th to the 75th percentile, while the horizontal divider marks the median. Mean and standard deviations are overlaid in black (0.87 ± 0.37 and 0.04 ± 0.02 for confined and dispersed respectively). These represent the spatial density of maxima, although, the density is obtained by projecting all maxima over the time span of the video. Hence, the spatial density of maxima per unit time can be obtained by divided by the total time of the video (30 s). Thus we obtain the unit of $\mu m^2/s$. (F) Data in E accounts for the density of maxima accumulated over the length of a video, without presenting any estimate for the persistence of a single maximum within the confinement areas. To quantify persistence, we look for the time interval between the appearance of at least one maximum inside a confinement area. The histogram, shows that a maximum is observed inside a confinement area every consecutive frame ($\sim 500\,ms$) with a probability of ~ 0.7. This implies that a maximum can be continuously detected inside a confinement zone for a period of atleast $30\,s$. The time limit of $30\,s$ arises out of chromatin motion which causes DNA bound spots to drift out of the plane in that time frame. (G) To summarize, we draw this histogram, which shows that the overall fraction of time for which a confinement area is occupied during the time span of $30\,s$. The mean obtained from this histogram is $\sim 65\%$.

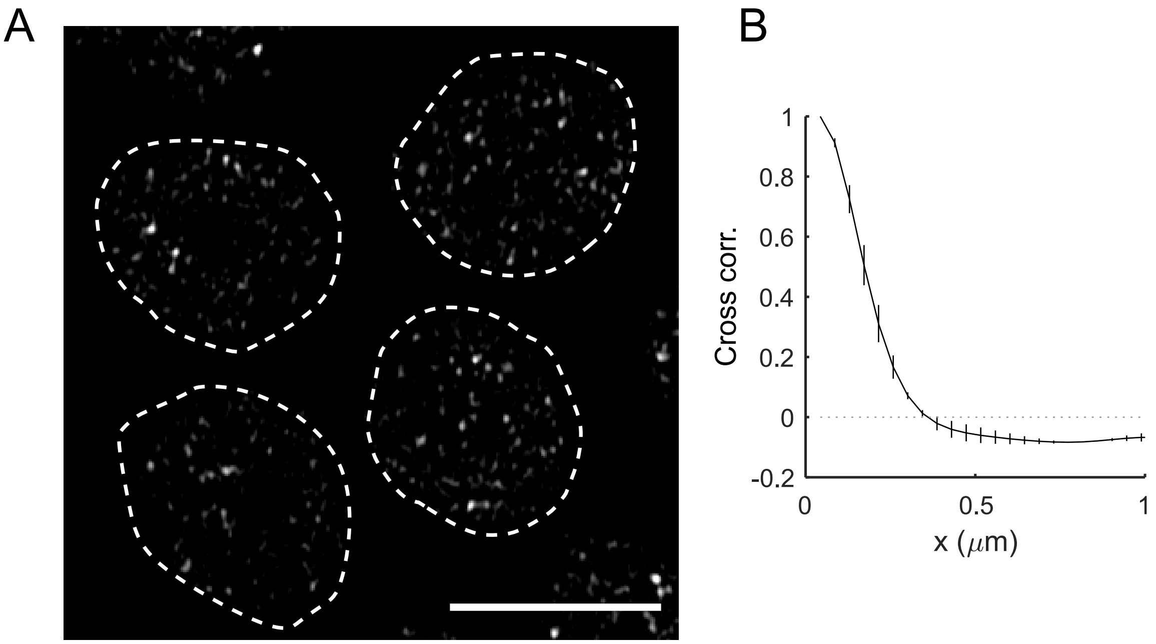

FIG. S5. **Bcd-GFP clusters in fixed embryos.** (A) Image shows cross section of nuclei expressing Bcd-GFP fixed using Formaldehyde. The dashed lines are guides to the eye for the nuclear boundaries. The scale bar is $5\mu m$ long. (B) Figure shows pixel cross correlation function (mean±std, n = 15 nuclei) on the nuclear pixels of a fixed Bcd-GFP embryo. Exponential fit to the plot gives a correlation length of 0.24 ± 0.01 μm, which is similar to the correlation length in live embryos.

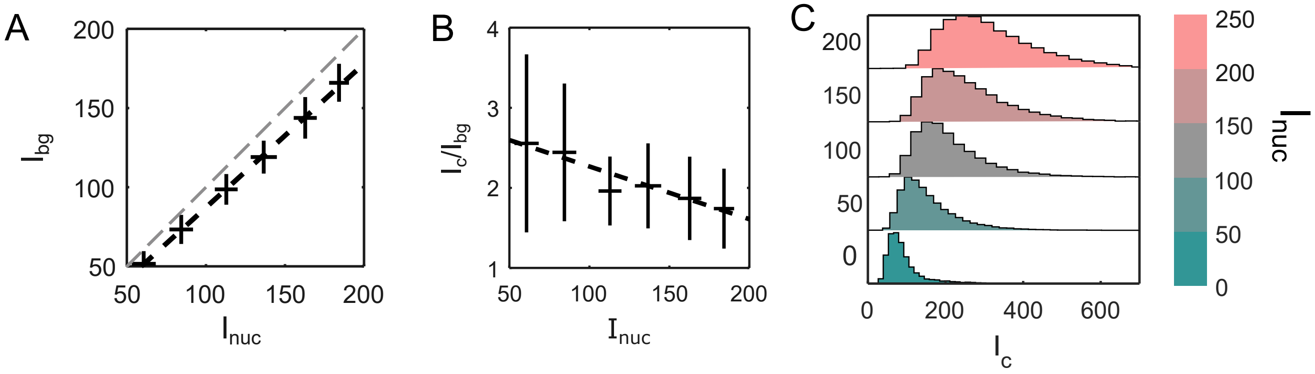

FIG. S6. **Molecular enrichment within clusters.** (A) Plot of average Bcd-GFP cluster background (I_{bg}) against average nuclear Bcd-GFP intensity ($R^2 = 0.95$). The dashed grey line represents $y = x$. (B) The nuclear average of the ratio of the peak cluster intensity to the respective background intensity (I_c/I_{bg}), plotted as a function of the average nuclear intensity I_{nuc}. The ratio is higher for lower nuclear concentrations. (C) Histograms showing the distribution of cluster peak intensities, I_c for different average nuclear intensity bin, I_{nuc}s. The color bar represents ranges of I_{nuc}

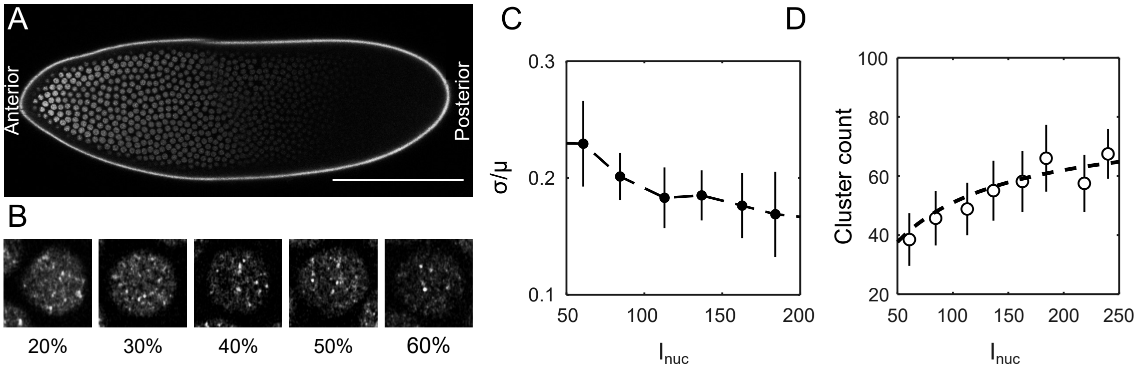

FIG. S7. **Cluster count.** (A) Cross-section of a *Drosophila* embryo in nuclear cycle #14 showing nuclei expressing Bcd-GFP. the imaging plane was near the surface of the embryo. The anterior and posterior ends of the embryo are marked. The higher GFP intensities in the anterior nuclei indicate a higher concentration of Bcd. The scale bar is 100 μm. (B) Left-to-right are zoomed images of cross-sections of individual nuclei expressing Bcd-GFP. The horizontal positions of the nuclei, measured from the anterior end are expressed as the percentage of embryo lengths below the respective panels. Images are $8 \times 8\,\mu$m in size. (C) The coefficient of variation (CV) of the cluster count per nucleus expressed as the standard deviation normalized to the mean values. Values are computed from pooled data (14 embryos, 2027 nuclei) discretized into nuclear Bcd-GFP intensity (I_{nuc}) bin. With an average CV of $< 20\%$, the cluster count displays remarkable reproduciblility across embryos. (E) To speculate about the nuclear concentration (I_{nuc}) dependence of the cluster counts, we employ an emperical relation (described in methods). The dashed line shows the fit of the cluster count data using that empirical relation.

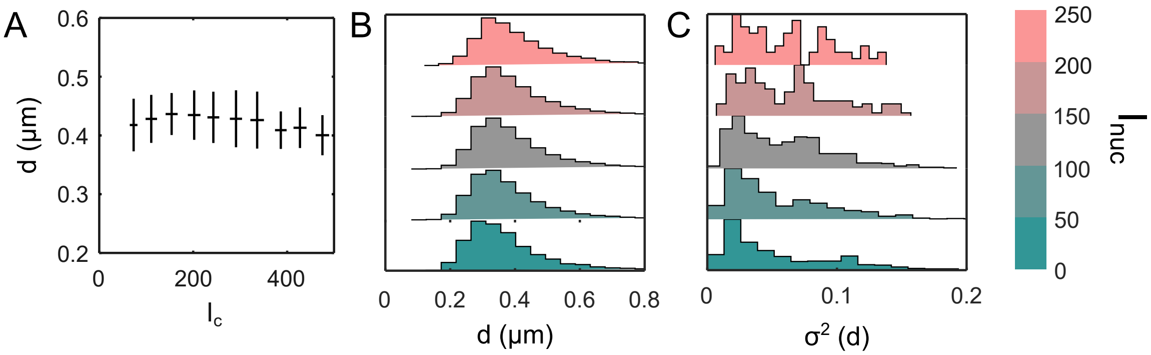

FIG. S8. **Cluster size.** (A) Is the cluster size dependent on the concentration of the cluster? To answer this, this figure shows the dependence of the cluster diameter, d on the cluster intensity, I_c. The cluster size, d is independent of I_{nuc}. (B) Clusters are sorted according to the I_{nuc} of their parent nucleus, and the histograms of cluster diameter, d are plotted for clusters belonging to various I_{nuc} range. The I_{nuc} range is indicated by the colorbar to the right of C. Interestingly, the histograms remain similar across a large range of I_{nuc}. This indicates that the cluster size is indeed independent of the nuclear concentration. (C) Here we visualize the variability of of the variance in cluster size within a nucleus. This figure shows the histograms of variances of cluster size per nucleus. The nuclei are sorted according to the range of I_{nuc}. (Colorbar to the right indicates the I_{nuc} range).

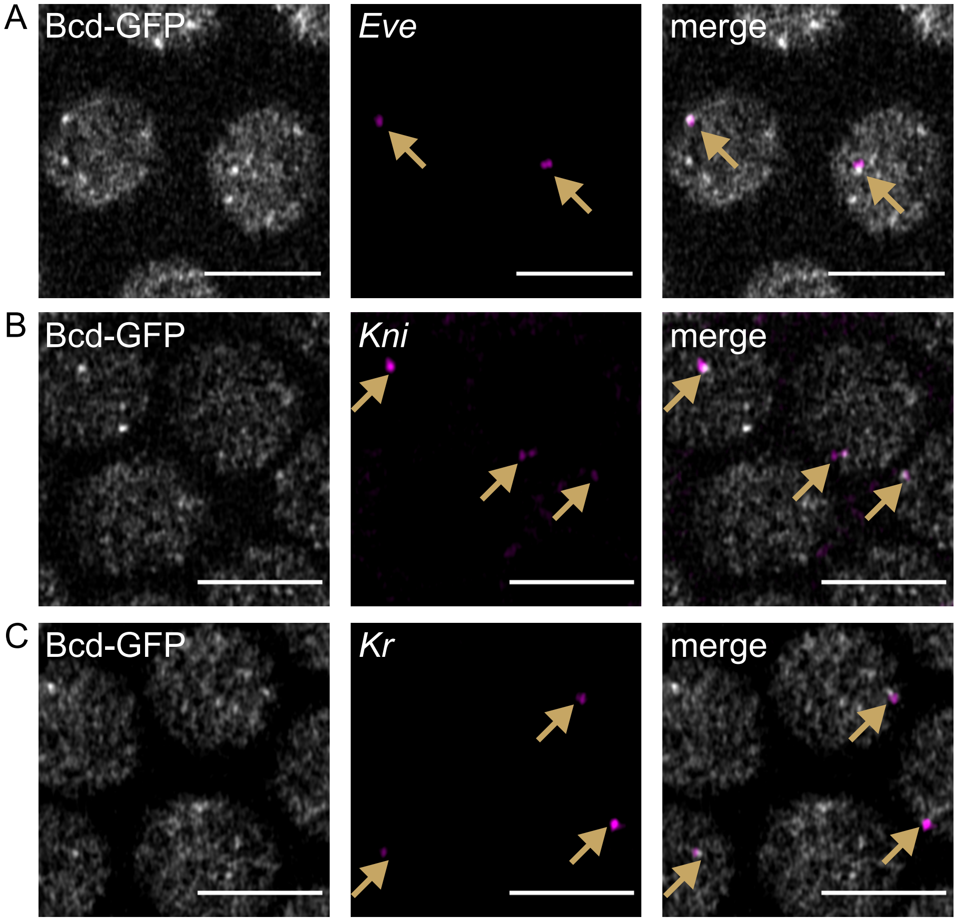

FIG. S9. **Accuracy of nuclear position determination using various cluster parameters.** (A) Natural logarithm of Bcd-GFP peak cluster intensity, I_c expressed as a function of fractional egg length x/L. The exponential decay constant extracted from linear fit is $\lambda = 0.32 \pm 0.03$ EL. (B) The Coefficient of Variation (CV) of I_c expressed as the coefficient of variation. The average CV is $\sim 30\,\%$. (C) The precision of nuclear position determination using the average I_c is $\sim 10\%$, which is equivalent to ~ 4 nuclear width. this shows that I_c is less precise in determination of the nuclear position that I_m. (D) Natural logarithm of Bcd-GFP peak cluster intensity, I_{bg} expressed as a function of fractional egg length x/L. The exponential decay constant extracted from linear fit is $\lambda = 0.25 \pm 0.02$ EL, which is statistically similar to the gradient of I_{nuc}. (E) The Coefficient of Variation (CV) of I_{bg} expressed as a function of x/L. The average CV is $< 20\,\%$. (F) The precision of nuclear position determination using the average I_{bg}. The precision is $\sim 4\,\%$, which is equivalent to ~ 1.5 nuclear width, which is equivalent to the precision in I_{nuc}. (G) Natural logarithm of Bcd-GFP peak cluster intensity, d expressed as a function of fractional egg length x/L. The exponential decay constant extracted from linear fit is $\lambda = 10.7 \pm 0.9$ EL. (H) The Coefficient of Variation (CV) of d expressed as a function of x/L. The average CV is $\sim$ 10 %. (I) The precision of nuclear position determination using the average d. The precision is $\sim 100\,\%$, which is equivalent to ~ 1 embryo length. This shows that d can not sense nuclear position.

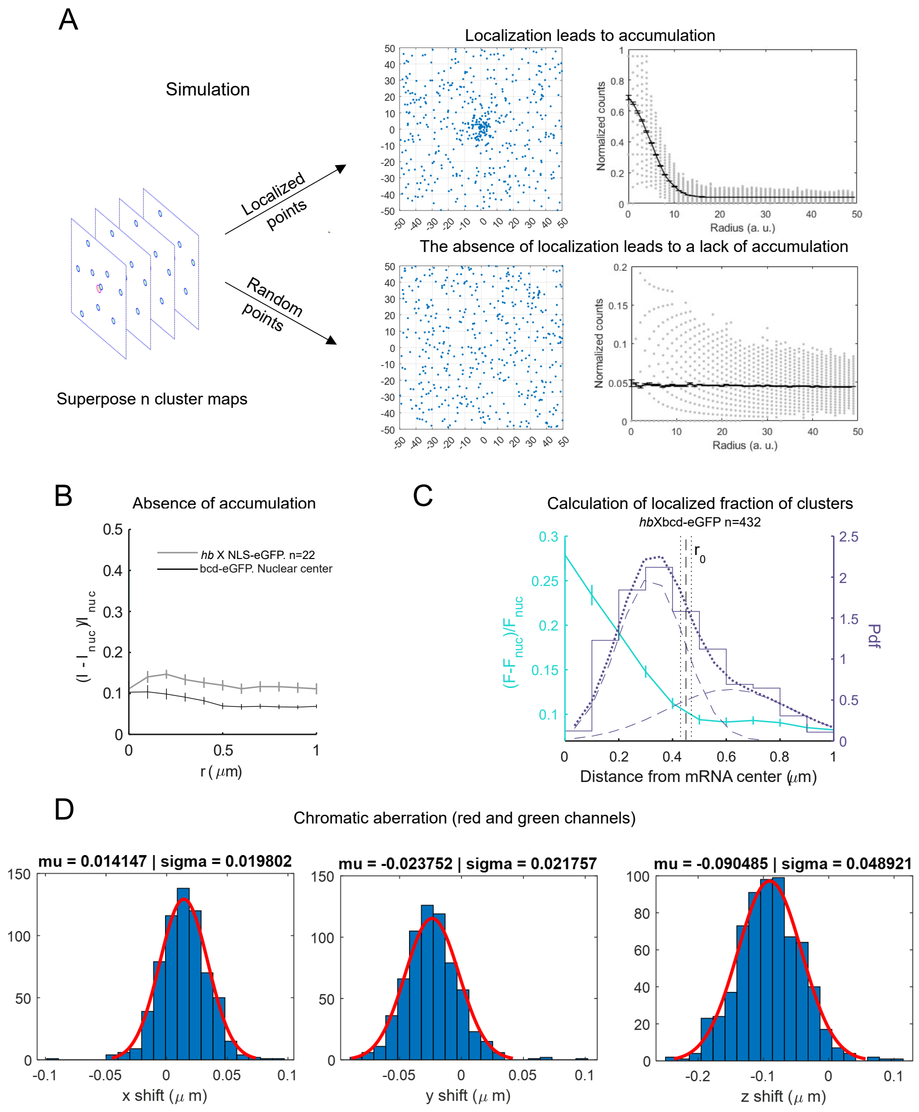

FIG. S10. **Accuracy of nuclear concentration determination using various cluster parameters.** (A) Total cluster intensity, I_m expressed as a function of nuclear Bcd-GFP intensity I_{nuc}. (B) The Coefficient of Variation (CV) of I_m expressed as a function of I_{nuc}. The average variability is $\sim 15\,\%$. (C) The precision of nuclear concentration (I_{nuc}) determination using the average I_c is 0.23 ± 0.07. However, the precision in concentration prediction is $\sim$ 15% for the anterior nuclei. (D) Peak cluster intensity, I_c expressed as a function of I_{nuc}. The slope of the linear fit is $slope = 1.19 \pm 0.10$ EL. (E) The CV of I_c expressed as a function of I_{nuc}. The average CV is $\sim 25\,\%$. (F) The precision of nuclear concentration (I_{nuc}) determination using the average I_c is 0.43 ± 0.20, equivalent to $\sim$ 40% error. Thus, I_c is less precise in determination of the I_{nuc} that I_m. (G) Background cluster intensity, I_{bg} expressed as a function of I_{nuc}. The slope of the linear fit is $slope = 0.99 \pm 0.02$ EL. (H) The CV of I_{bg} expressed as a function of I_{nuc}. The average CV is $\sim 10\,\%$, making it a highly reproducible quantity, similar to I_{nuc}. (I) The error in estimating of nuclear concentration (I_{nuc}) using the average I_{bg} is $<$ 10% in the anterior. (J) The cluster diameter, d expressed as a function of nuclear Bcd-GFP intensity I_{nuc}. (K) The CV of d expressed as the coefficient of variation. The average variability is $< 10\,\%$. (L) The precision of nuclear concentration (I_{nuc}) determination using the average d is $>$ 100%, making cluster size an extremely imprecise metric for estimating I_{nuc}.

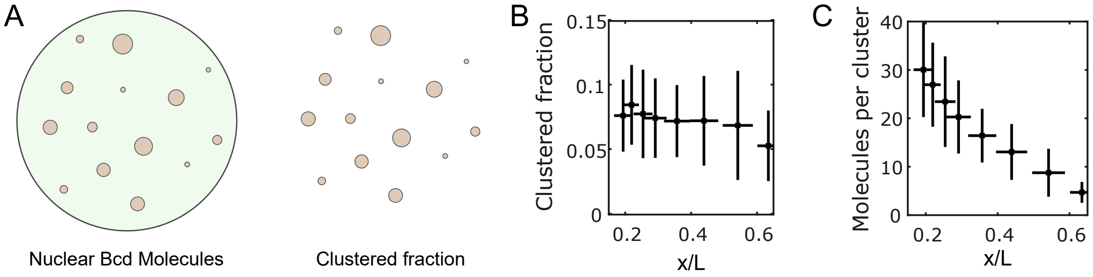

FIG. S11. **Estimation of the number of molecules per cluster.** (A) A cartoon depicting the broad classification of distribution of the Bcd molecules in the nucleus. The green depicts the freely diffusing Bcd molecules, while the brown (also separately shown on the right) shows the clustered fraction. (B) Figure shows the fraction of Bcd molecules in the clustered fraction per nucleus ($mean \pm std$) as a function of the nuclear position in the embryo. It must be noted that the clustered fraction is defined by the limits of detection in this study. (C) The average molecules of Bcd per cluster ($mean \pm std$) is plotted as a function of the nuclear position in the embryo (Methods). The average number of molecules drops from about 30 at the anterior to 5 in the posterior half of the embryo. This also denotes the lower limit of detection, setting the limit to ~ 5 molecules per cluster.

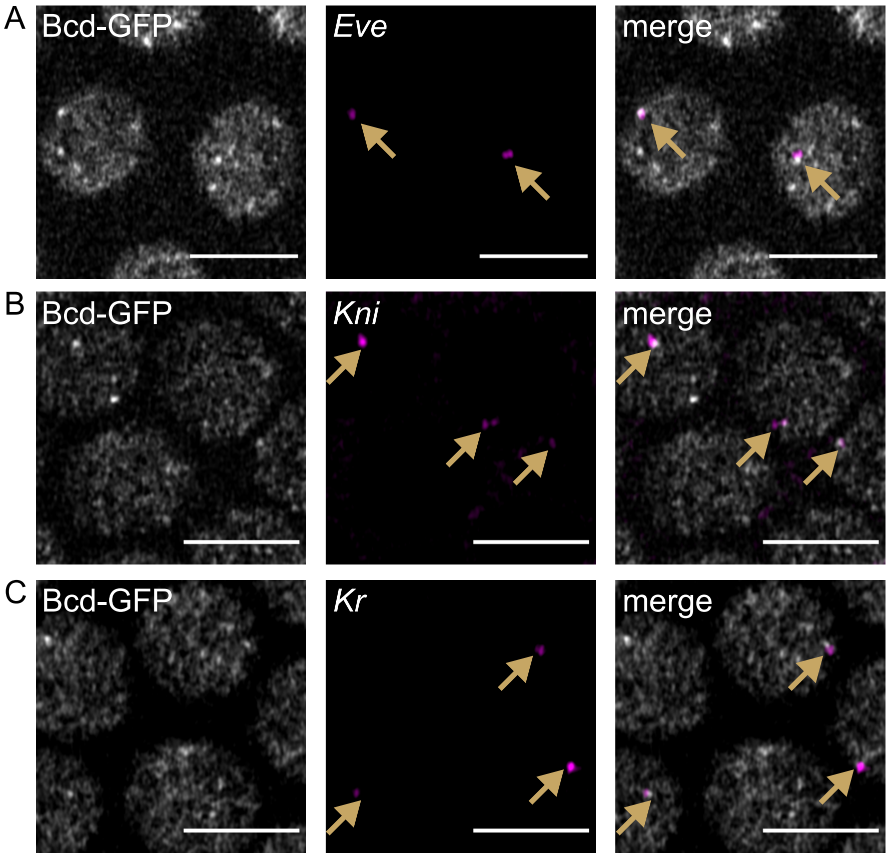

FIG. S12. **Micrographs of Bcd clusters colocalizing with nascent transcription hotspots.** The left panels show the raw images of nuclei expressing Bcd-GFP. The middle panels show the same nuclei expressing (A) *Eve*, (B) *Kni* and (C) *Kr*. The right panels shows the two images from the left and the middle overlaid. Arrows indicate the transcriptional hotspots.

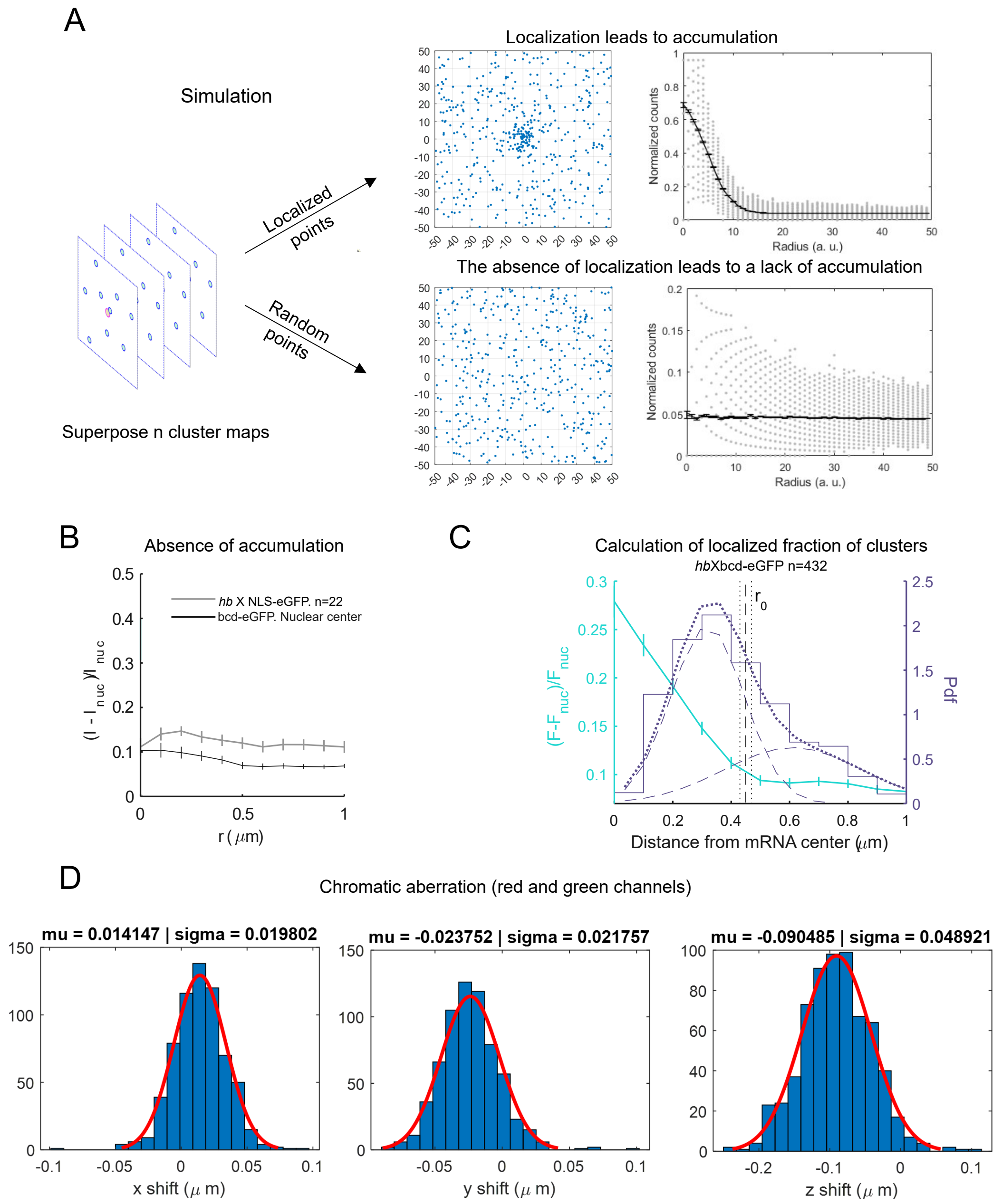

FIG. S13. **Localization of Bcd cluster with the nascent transcription hotspot.** (A) (Left) Cartoons representing a nascent transcription hotspot (pink) and TF molecules (blue). (Right) With a nascent transcription hotspot at the center of the simulation window, TF molecules preferentially accumulate near the origin, (TOP) giving a Gaussian profile for the radial cluster density. The size of the Gaussian function (FWHM) gives the spread of the accumulation. Molecules appearing within this limit can be considered to be coupled to the hotspot at the origin in some capacity. (Bottom) Absence of a transcription hotspot at the origin leads to a random distribution of molecules, yielding a flat profile (RIGHT). Examples of this can be seen in B. (B) NLS-eGFP does not accumulate at the *hb* transcription site (gray). Also, Bcd-GFP does not accumulate at a random site in the nucleus (nuclear center). (C) Since the TF accumulation radius defines in some sense the "limit of influence" of a target gene, any TF cluster appearing within this limit should be considered coupled to the gene. To test this hypothesis, the histogram of the distance of the Bcd-GFP clusters nearest to *hb* transcription hotspot is plotted. The histogram is then fitted with a double Gaussian, and the position of intersection of the two Gaussian kernels is compared with the "limit" obtained earlier. The two quantities are within $\sim 50\,nm$ of each other. (D) The three plots show the histograms of shifts in the intensity-weighted centers of polystyrene beads measured in two color channels, red and green. A Gaussian fit to each histogram gives the corresponding σ which serves as a measure of chromatic aberration along each image axis. All three shifts are sub-pixel.

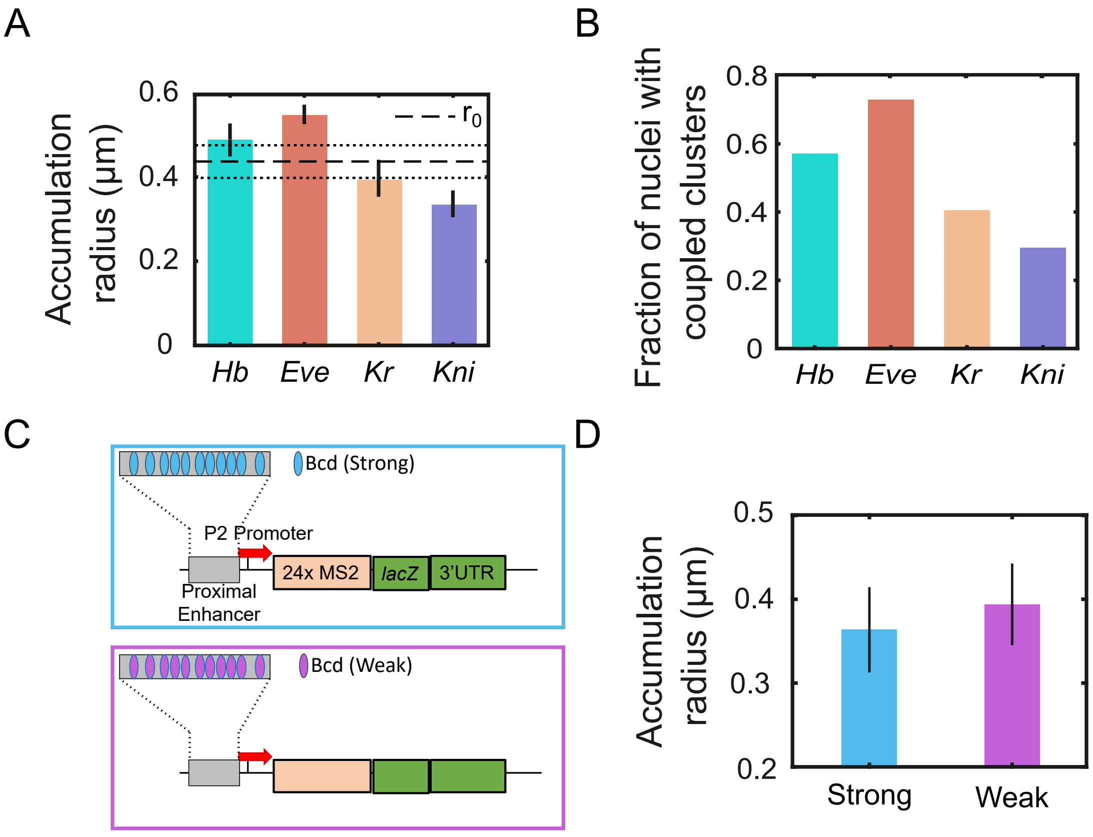

FIG. S14. **Cluster coupling characteristics with the target genes.** (A) Bars representing 2σ from the Half normal fits of Bcd-GFP accumulation (see Methods) for each target gene (labeled on the x axis). All bars indicate standard errors. The horizontal line shows the average (dashed) and standard error bounds(dotted) from the entire dataset, calculated via bootstrapping. The average radius is also shown in C by a vertical dashed line. (B) The fraction of nuclei in which expressing target genes are associated with a coupled cluster. The corresponding genes are labeled on the x axis. (C) Schematic shows the two constructs, one driven by an enhancer with strong Bcd binding sites, and the other with an enhancer composed of weak Bcd binding sites. (D) Bars representing 2σ from the Half normal fits of Bcd-GFP accumulation for the two constructs shown in C.

II. SUPPLEMENTAL TABLES

Fly line	Source
hb BAC<MS2	Bothma *et al.*[54]
kni BAC<MS2	Bothma *et al.*[54]
eve MS2	Chen *et al.*[47]
kr MS2	El-Sherif *et al.*[55]
bnk MS2	This work
P2 (Strong) MS2	This work
P2 (Weakg) MS2	This work

TABLE I. **List of MS2 stem loop fly lines.**
